# Tetramerization is essential for the enzymatic function of the *Pseudomonas aeruginosa* virulence factor UDP-glucose pyrophosphorylase

**DOI:** 10.1128/mbio.02114-23

**Published:** 2024-03-12

**Authors:** Larissa Dirr, Sven Cleeves, Isabel Ramón Roth, Linghui Li, Timm Fiebig, Thomas Ve, Susanne Häussler, Armin Braun, Mark von Itzstein, Jana I. Führing

**Affiliations:** 1 Institute for Glycomics, Gold Coast Campus, Griffith University, Gold Coast, Queensland, Australia; 2 Fraunhofer Institute for Toxicology and Experimental Medicine ITEM, Member of Fraunhofer International Consortium for Anti-Infective Research (iCAIR), Hannover, Germany; 3 Biomedical Research in Endstage and Obstructive Lung Disease (BREATH), German Center for Lung Research (DZL), Hannover, Germany; 4 Institute of Clinical Biochemistry, Hannover Medical School, Hannover, Germany; 5 Department of Molecular Bacteriology, Helmholtz Centre for Infection Research, Braunschweig, Germany; 6 Institute for Molecular Bacteriology, TWINCORE, Centre for Experimental and Clinical Infection Research, Hannover, Germany; 7 Department of Clinical Microbiology, Copenhagen University Hospital - Rigshospitalet, Copenhagen, Denmark; 8 Cluster of Excellence RESIST (EXC 2155), Hannover Medical School, Hannover, Germany; 9 Institute of Immunology, Hannover Medical School, Hannover, Germany; Massachusetts General Hospital, Boston, Massachusetts, USA

**Keywords:** drug targets, *Pseudomonas aeruginosa*, protein structure-function, virulence factors, lipopolysaccharide, virulence, opportunistic infections, antibiotic resistance, lung infection, precision-cut lung slices, UDP-glucose pyrophosphorylase, *galU*, oligomerization

## Abstract

Multidrug-resistant bacteria such as the opportunistic pathogen *Pseudomonas aeruginosa*, which causes life-threatening infections especially in immunocompromised individuals and cystic fibrosis patients, pose an increasing threat to public health. In the search for new treatment options, *P. aeruginosa* uridine diphosphate-glucose pyrophosphorylase (PaUGP) has been proposed as a novel drug target because it is required for the biosynthesis of important virulence factors and linked to pathogenicity in animal models. Here, we show that UGP-deficient *P. aeruginosa* exhibits severely reduced virulence against human lung tissue and cells, emphasizing the enzyme’s suitability as a drug target. To establish a basis for the development of selective PaUGP inhibitors, we solved the product-bound crystal structure of tetrameric PaUGP and conducted a comprehensive structure-function analysis, identifying key residues at two different molecular interfaces that are essential for tetramer integrity and catalytic activity and demonstrating that tetramerization is pivotal for PaUGP function. Importantly, we show that part of the PaUGP oligomerization interface is uniquely conserved across bacterial UGPs but does not exist in the human enzyme, therefore representing an allosteric site that may be targeted to selectively inhibit bacterial UGPs.

**IMPORTANCE:**

Infections with the opportunistic bacterial pathogen *Pseudomonas aeruginosa* are becoming increasingly difficult to treat due to multidrug resistance. Here, we show that the enzyme uridine diphosphate-glucose pyrophosphorylase (UGP) is involved in *P. aeruginosa* virulence toward human lung tissue and cells, making it a potential target for the development of new antibacterial drugs. Our exploration of *P. aeruginosa* (Pa)UGP structure-function relationships reveals that the activity of PaUGP depends on the formation of a tetrameric enzyme complex. We found that a molecular interface involved in tetramer formation is conserved in all bacterial UGPs but not in the human enzyme, and therefore hypothesize that it provides an ideal point of attack to selectively inhibit bacterial UGPs and exploit them as drug targets.

## INTRODUCTION

Bacterial multidrug resistance poses a growing threat to global health and has prompted renewed efforts toward the discovery and exploitation of novel drug targets. The enzyme uridine diphosphate (UDP)-glucose pyrophosphorylase (UGP, EC 2.7.7.9) occupies a key position in UDP-sugar metabolism as it converts glucose-1-phosphate (Glc-1-P) and uridine triphosphate (UTP) to inorganic pyrophosphate (PP_i_) and UDP-glucose (UDP-Glc); the latter, in turn, can be converted into UDP-glucuronic acid, UDP-galactose, and UDP-galacturonic acid. Bacteria utilize these (and other) nucleotide sugars as activated glycosyl donors for the synthesis of virulence factors such as lipopolysaccharides (LPS), capsular polysaccharides (CPS), and exopolysaccharides (EPS) (1); accordingly, UGP was shown to be crucial for the virulence of various clinically important bacterial pathogens, including *Streptococcus pneumoniae* (2), *Escherichia coli* (3), *Vibrio cholerae* (4), *Klebsiella pneumoniae* (5), and *Pseudomonas aeruginosa* (6).

*P. aeruginosa* is a Gram-negative opportunistic pathogen causing a wide range of severe acute and chronic diseases, especially in immunocompromised or immunosuppressed individuals, and has emerged as a major cause of healthcare-associated infections, aided by its capability to form biofilms that facilitate the colonization of indwelling medical devices such as catheters and endotracheal tubes (7). The bacterium frequently causes bacteremia, endocarditis, meningitis, keratitis, and diabetic foot ulcers as well as infections of the urinary or gastrointestinal tract, burn wounds, and surgery sites. Perhaps most importantly, *P. aeruginosa* is notorious for causing severe pneumonia in individuals suffering from chronic obstructive pulmonary disease or cystic fibrosis (CF), where it is the predominant pathogen infecting adult CF patients and a leading cause of respiratory failure and mortality (8). To date, no approved *P. aeruginosa* vaccines are available (9), and infections become increasingly difficult to treat due to innate antibiotic resistances caused by low outer membrane permeability and efflux systems, in addition to which *P. aeruginosa* readily acquires and develops further resistances (10). A recent meta-analysis on antibiotic resistance patterns of *P. aeruginosa* infections in CF patients (11) found alarming resistance rates of up to 67% for some antibiotics, resulting from repeated antibiotic treatment (12). Although there is some hope that the relatively new modulator therapies aimed at restoring cystic fibrosis transmembrane conductance regulator (CFTR) function could also reduce the bacterial burden of typical CF pathogens, and some studies have found modulator therapies to decrease *P. aeruginosa* prevalence in CF lungs [reviewed in reference (13)], conflicting results have been reported, and the persistence of the observed effects is still unclear. Therefore, careful management of existing therapeutic regimens as well as the development of new drugs for the treatment of multi-resistant *P. aeruginosa* infections still remain an urgent priority.

LPS is a major virulence factor of *P. aeruginosa,* which protects the bacterium from host defense mechanisms, mediates interactions with host factors, other bacterial cells as well as antibiotics, and triggers inflammatory responses causing endotoxicity (14). LPS consists of a lipid A anchoring it to the outer bacterial membrane; a branched core oligosaccharide; and the O-polysaccharide or O-antigen (15), which confers protection from phagocytosis and complement-mediated killing (16) and is involved in motility and surface attachment (17). While the O-antigen differs in monosaccharide composition and/or linkage between the 20 *P*. *aeruginosa* serotypes, the core oligosaccharide composition is conserved and contains Glc as the predominant sugar (15), making UDP-Glc an essential precursor for LPS synthesis. Accordingly, UGP-deficient mutants of *P. aeruginosa* display a truncated LPS core lacking Glc (18) and are consequently devoid of O-antigen (i.e., LPS-rough phenotype) (19). Loss of UGP function was shown to cause increased sensitivity toward human serum, strongly attenuated virulence in a murine corneal infection model, and 1–2 log higher LD_50_ doses in an acute murine pneumonia model, associated with reduced bacterial burden in the lungs and reduced systemic spread (6). Beyond its involvement in the synthesis of the LPS core, UDP-Glc is a precursor of UDP-aminoarabinose, required for an inducible modification of the LPS lipid A frequently found in chronic *P. aeruginosa* lung infections of CF patients (20), which confers resistance to cationic antimicrobial peptides (CAMPs) (21). Mutational inactivation of UGP has been shown to increase *P. aeruginosa* sensitivity to polymyxin B, colistin, indolicidin, and other CAMPs (22). Furthermore, in some *P. aeruginosa* strains, UGP is important for the production of secreted biofilm matrix (sometimes referred to as capsule), which facilitates the colonization of abiotic surfaces and mediates antibiotic tolerance, as UDP-Glc and its derivative UDP-galactose are required for the synthesis of Psl (23, 24), one of the three major EPS synthesized by *P. aeruginosa*. The common laboratory strain PAO1 primarily utilizes Psl as EPS (25), and inactivation of UGP was shown to result in the complete loss of Psl (24) as well as sensitization to the biofilm inhibitor Polysorbate 80 (26).

Similar to *P. aeruginosa*, loss of UGP function has been shown to result in phenotypes such as reduced adherence and colonization, impaired motility and biofilm formation, as well as increased sensitivity to serum, antimicrobials, and environmental stress factors in various bacterial pathogens. Consequently, bacterial UGPs have been proposed as attractive new “anti-virulence” drug targets, which might have the added benefit of exerting less selective pressure toward resistance development since the target is non-essential (27, 28). In contrast, UGP of the human host is an essential enzyme, as it provides substrates for crucial processes such as glycogen synthesis, glycosylation, glucuronidation, and glycoprotein folding control; accordingly, no viable loss-of-function mutants have been described (29). Although bacterial and eukaryotic UGPs are considered to be evolutionarily unrelated (1), both utilize an ordered sequential Bi Bi mechanism with UTP binding preceding that of Glc-1-P, product formation occurring via an S_N_2 nucleophilic attack, and PP_i_ exiting the active site prior to UDP-Glc (1, 30). In line with their common function, all UGPs share a similar Rossman-like active site fold; thus, the development of bacterial UGP inhibitors should avoid targeting the catalytic center since such compounds could cross-react with the essential human UGP (HsUGP). Therefore, our work aims at identifying unique structural and/or functional features that can be exploited for selective inhibition of the bacterial enzyme. In this regard, mechanistic differences linked to the respective quaternary structure are of specific interest: fungal and animal UGPs form octamers (31–35), and we previously demonstrated that octamerization is required for HsUGP function (29, 36). In contrast, plant UGPs are active monomers but appear to be regulated by oligomerization (37–40), whereas protozoan UGPs form exclusively active monomers (41, 42). Bacterial UGPs are described to form tetramers or dimers (1, 43); however, it has not been conclusively investigated whether oligomerization promotes or impairs their activity.

Toward exploiting *P. aeruginosa* UGP (PaUGP) as a drug target, here we studied the enzyme’s role in virulence against human lung tissue and cells, solved its product-bound X-ray crystal structure, identified catalytically important active site residues, and analyzed the relationship between oligomerization and activity by mutagenesis. We show that, compared to wild-type (WT) *P. aeruginosa*, a UGP-deficient strain possesses inherently reduced cytotoxicity toward *ex vivo* human lung tissue and *in vitro* cultured human lung epithelial cells. Our structure-function studies identified a network of amino acids involved in the formation of the native, active PaUGP tetramer, including three key residues whose mutation caused dissociation linked to loss of activity, clearly demonstrating that PaUGP function depends on tetramerization. Our findings suggest that targeting an oligomerization interface, which is conserved across bacterial UGPs but absent from the human enzyme, can facilitate selective inhibition of not only PaUGP but also bacterial UGPs in general. In conclusion, this study provides a new direction in the structure-guided development of novel antibiotics targeting *P. aeruginosa—*and potentially further bacterial pathogens—that would respond to unmet clinical needs.

## RESULTS

### *In vitro* growth of PAO1 is unaffected by the loss of UGP function

Toward validating PaUGP as a drug target, we first examined the effects of the loss of UGP function on *P. aeruginosa* viability and growth *in vitro*. Therefore, we obtained a PAO1 mutant strain in which the *galU* gene, encoding UGP, was disrupted by transposon insertion (*galU^−^*) (44) and compared its growth kinetics to WT PAO1 over 24 h in nutrient-rich lysogeny broth (LB) medium as well as the two cell culture media used in the infection studies described below. Growth of both strains was similar or identical in all media tested (Fig. S1), indicating that UGP is dispensable for PAO1 survival and growth *in vitro*.

### PaUGP is dispensable for the colonization of Calu-3 cells *in vitro* and human lung tissue *ex vivo*

**Fig 1:**
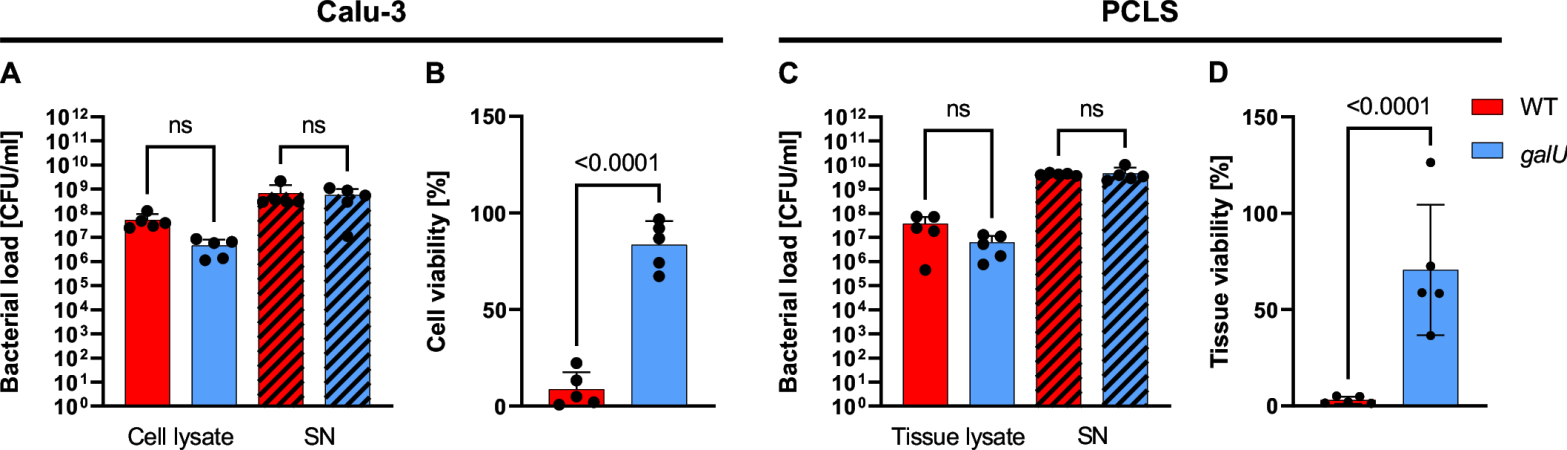
Bacterial loads in PAO1 WT and *galU*^−^-infected (**A**) Calu-3 and (**C**) human PCLS lysates and culture supernatants (SN, hatched bars), and relative viability of (**B**) Calu-3 cells and (**D**) human PCLS, 24 h after infection. Viability was measured by calcein fluorescence and is given as a percentage of the respective uninfected control (≙100%). Data are from five individual experiments. Error bars indicate mean ± SD; ns, not significant. One-way ANOVA with Tukey’s multiple comparisons test was performed. Adjusted *P*-values are displayed above the bars.

To assess whether UGP functionality influences PAO1’s ability to colonize and grow in the presence of host-derived lung cells and tissue, we quantified cell-/tissue-associated (in the lysate) and planktonic (in the supernatant) bacteria 24 h after infecting Calu-3 human lung epithelial cells or human precision-cut lung slices (PCLS) with PAO1 WT or the *galU^−^* mutant. PCLS are 200–300 µm thick slices of native human lung tissue and contain physiologically and immunologically relevant cell types—including airway and alveolar epithelial cells (ciliated, goblet, and club cells), endothelial cells, tissue-resident macrophages, and neuronal cells—surrounded by extracellular matrix. No significant differences in bacterial loads were observed between PAO1 WT and *galU^−^*-infected lung cells or PCLS (Fig. 1A and C; Table S1), indicating a neglectable role of UGP in colonization.

### PAO1 *galU^−^* shows strongly reduced virulence in Calu-3 cells and human lung tissue *ex vivo*

To determine the effect of loss of UGP function on PAO1 virulence, we quantified the viability of Calu-3 cells and human PCLS 24 h after infection with WT or the *galU^−^* mutant by measuring calcein fluorescence. Whereas the average viability of Calu-3 cells infected with WT was reduced to approximately 9%, *galU^−^*-infected cells retained circa 83% viability (Fig. 1B). Similarly, PAO1 WT caused strong cytotoxicity in human PCLS 24 h after infection, reducing tissue viability to approximately 3% of the uninfected controls, whereas *galU^−^*-infected human PCLS retained circa 71% viability (Fig. 1D).

### PAO1 *galU*^−^ triggers lower pro-inflammatory cytokine response

Since *P. aeruginosa* infection should evoke a robust innate immune response that is in part caused by LPS in the bacterial outer membrane, we asked whether PAO1 WT and *galU*^−^ would trigger qualitatively and/or quantitatively different inflammatory responses. To this end, we quantified 10 pro-inflammatory cytokines in Calu-3 and PCLS supernatants by multiplex ELISA. Overall, a high variability in cytokine release could be observed in PCLS, while the response of Calu-3 cells was more uniform. In both systems, only TNF-α and IL-6 were substantially increased upon PAO1 infection. Interestingly, infection of Calu-3 cells with WT triggered a significantly higher secretion of TNF-α and IL-6 than *galU*^−^ (Fig. S2).

### Pyocyanin production is decreased in PAO1 *galU^−^*

**Fig 2:**
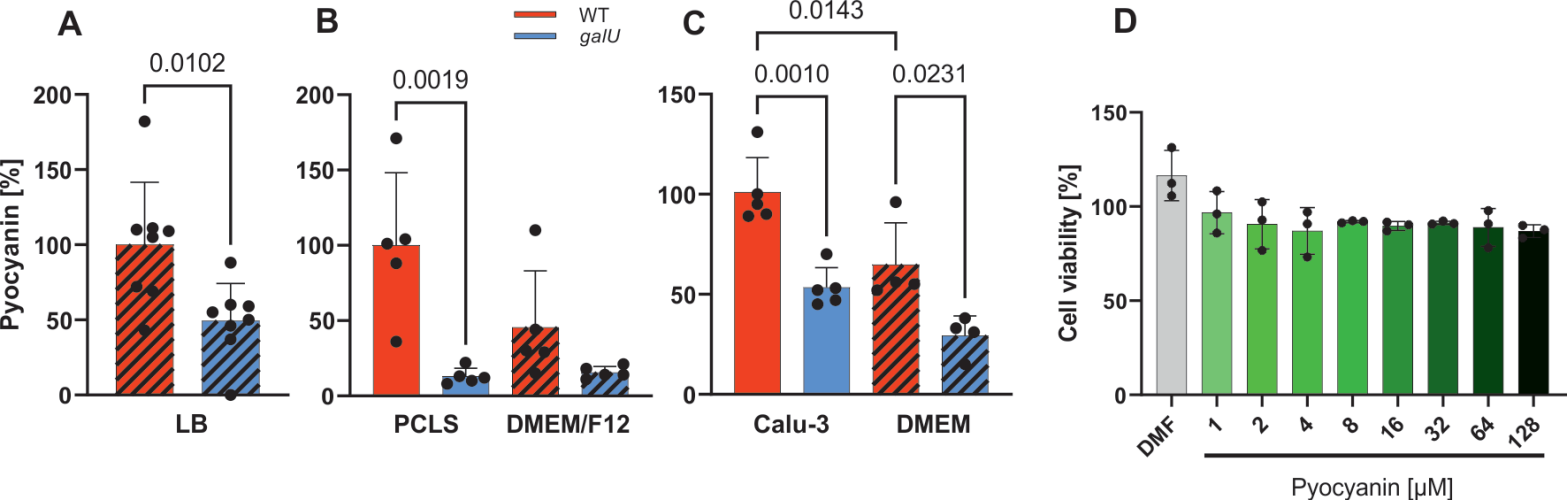
Pyocyanin secretion by WT and *galU*^−^ PAO1 and the effects of pyocyanin on Calu-3 cell viability. Relative pyocyanin levels in (**A**) overnight cultures (LB medium), (**B**) culture supernatants of infected PCLS, or (**C**) culture supernatants of infected Calu-3 cells, or the respective culture media alone (hatched bars). Pyocyanin content was normalized to bacterial load and is expressed as the percentage of the respective WT sample. Data are from at least four individual experiments. Error bars indicate mean ± SD. Unpaired *t*-test was performed for overnight cultures. One-way ANOVA with Tukey’s multiple comparisons test was performed for PCLS and Calu-3 data. Adjusted *P*-values are displayed above the bars. (**D**) Calu-3 cell viability after 24-h incubation with increasing concentrations of pyocyanin was quantified by calcein fluorescence and is given as the percentage of the medium-only control. Data are from three individual experiments. Error bars indicate mean ± SD. Dimethylformamide (DMF) was used as a solvent control for pyocyanin.

Owing to the pigment pyocyanin, cultures and cell/PCLS supernatants of WT PAO1 showed a characteristic blue-green color, which was visibly and quantifiably reduced in *galU*^−^ cultures and supernatants (Fig. 2A through C; Fig. S3). Since pyocyanin is a known contributor to *P. aeruginosa* virulence (45), we asked whether the higher pyocyanin concentration (quantified as 4.2 µM for PAO1 WT vs 2.15 µM for *galU*^−^ in the supernatants of Calu-3 cells) caused the higher cytotoxicity of the WT. However, treatment of uninfected Calu-3 cells with pyocyanin in concentrations up to 128 µM did not induce any detectable cytotoxicity (Fig. 2D).

### PAO1 *galU*^−^ exhibits reduced *in vitro* motility

**Fig 3:**
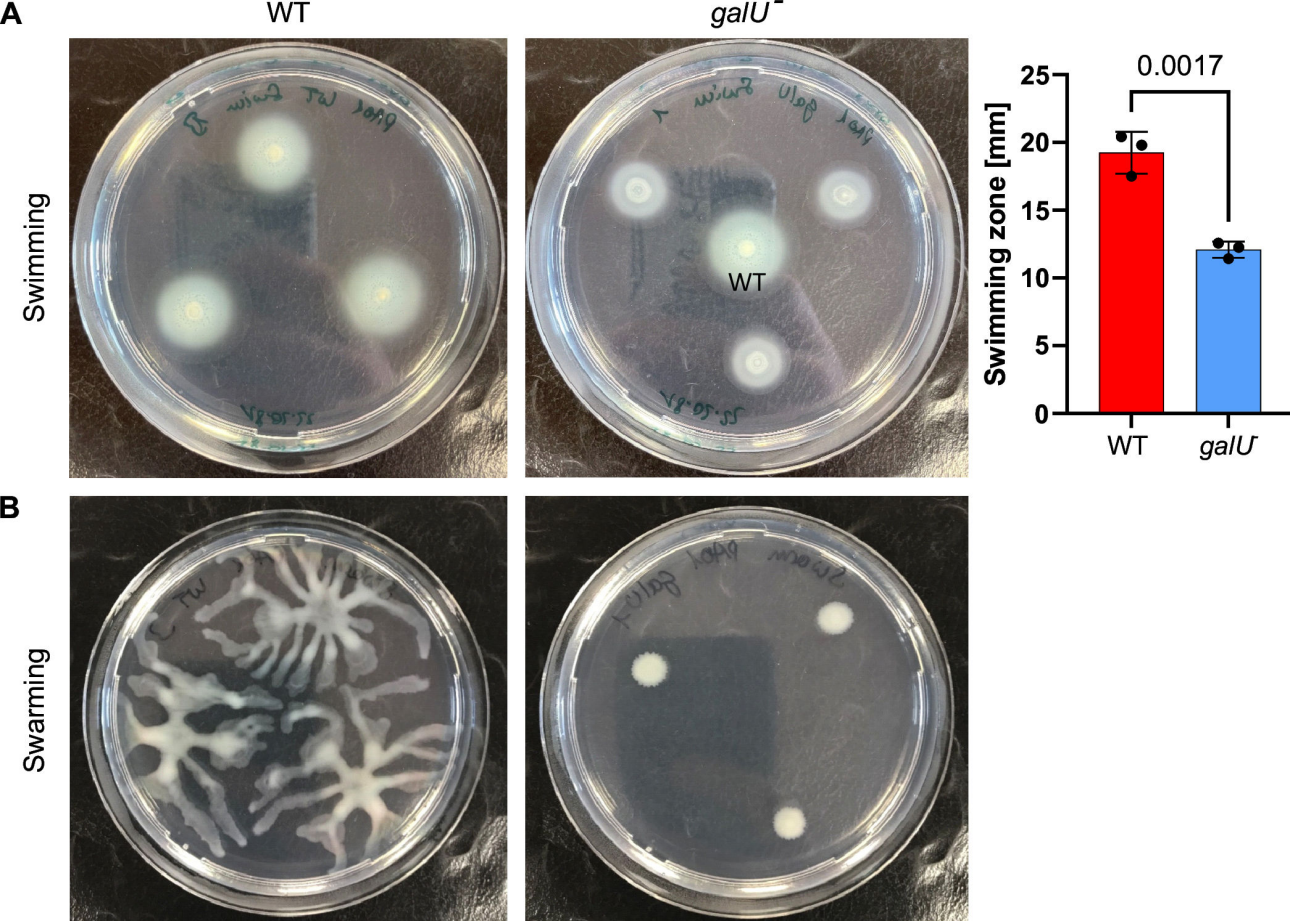
(**A**) Swimming and (**B**) swarming motility phenotypes of PAO1 WT and *galU*^−^. Representative images from three individual experiments are shown. Error bars indicate mean ± SD. Statistical significance was determined by unpaired *t*-test.

Since LPS is known to be involved in bacterial adhesion and motility, we asked whether the lack of UGP function impacts *P. aeruginosa* motility *in vitro*. Interestingly, swimming zones of PAO1 *galU*^−^ were significantly smaller than those of WT (Fig. 3A). Moreover, while WT showed extensive swarming behavior, almost no swarming was observed for *galU*^−^ (Fig. 3B).

### Recombinant production and characterization of PaUGP

Having demonstrated the importance of PaUGP for *P. aeruginosa* virulence in human lung cells and tissue, we aimed to structurally and functionally characterize the drug target *in vitro* to provide a basis for the rational development of selective PaUGP inhibitors. Toward this goal, we expressed and purified recombinant, enzymatically active Strep-tagged PaUGP. Size exclusion chromatography (SEC) showed a single oligomeric species with a calculated mass of ca. 130 kDa (Fig. S4A), corresponding to a tetramer of the 33 kDa protein identified by SDS-PAGE and subsequent Western blot analysis (Fig. S4B). These findings are in line with most publications describing tetrameric assemblies for bacterial UGPs (46–51). The addition of substrates (UTP and/or Glc-1-P) or product (UDP-Glc) did not affect oligomerization. In blue native (BN)-PAGE, PaUGP likewise migrated as a tetramer but showed mild, unspecific dissociation (see Fig. 6 below), likely due to differences in buffer composition.

### Crystal structure of PaUGP in complex with UDP-Glc

The crystal structure of the PaUGP*UDP-Glc complex was determined by molecular replacement using *Helicobacter pylori* (Hp) UGP (Protein Data Bank, PDB ID: 3JUK) (48) as a search model. The crystals have the symmetry of monoclinic space group P12_1_1 and diffracted to 2.9 Å resolution. Data processing and refinement statistics are listed in Table S2. The crystal structure comprises residues 1–279 of PaUGP, of which we were able to successfully model residues 1–275; the C-terminal residues were not modeled due to weak electron density. Each PaUGP subunit contains one UDP-Glc molecule and one Mg^2+^ ion in the active site (Fig. S5B). PaUGP and HpUGP are very similar with 1.28 Å root mean square deviation between 271 Cα atoms of protein. In HpUGP, two additional Mg^2+^ ions were found; however, they do not appear to be of biological relevance (48). The crystals of PaUGP contain eight molecules per asymmetric unit; however, through analysis of crystal contacts, it was possible to reassemble the biologically active homotetramer as seen in HpUGP (48).

### Subunit and active site structure

Each PaUGP subunit contains an open twisted central β-sheet surrounded by α-helices on both sides with a total of 12 α-helices and 12 β-strands (Fig. S5A), resembling the Rossman-fold characteristic for nucleotide-binding enzymes. We identified 13 residues presumably participating in substrate/product binding and/or catalysis (Fig. S5B), mutated them to alanine, and examined the oligomerization state and *in vitro* enzymatic activity compared to the WT enzyme, which was defined as 100% (Fig. S5C). Apart from G107A, all mutant enzymes could be obtained as soluble proteins in purities comparable to WT (Fig. S6A). Mutation to alanine did not affect the oligomerization state, but in most cases abolished enzymatic activity almost completely (Fig. S5C and S7), supporting the functional importance of the selected residues. A detailed description of the individual active site residues’ proposed roles and species comparisons is provided in the Supplemental Discussion.

### Intermolecular interactions in the PaUGP tetramer

**Fig 4:**
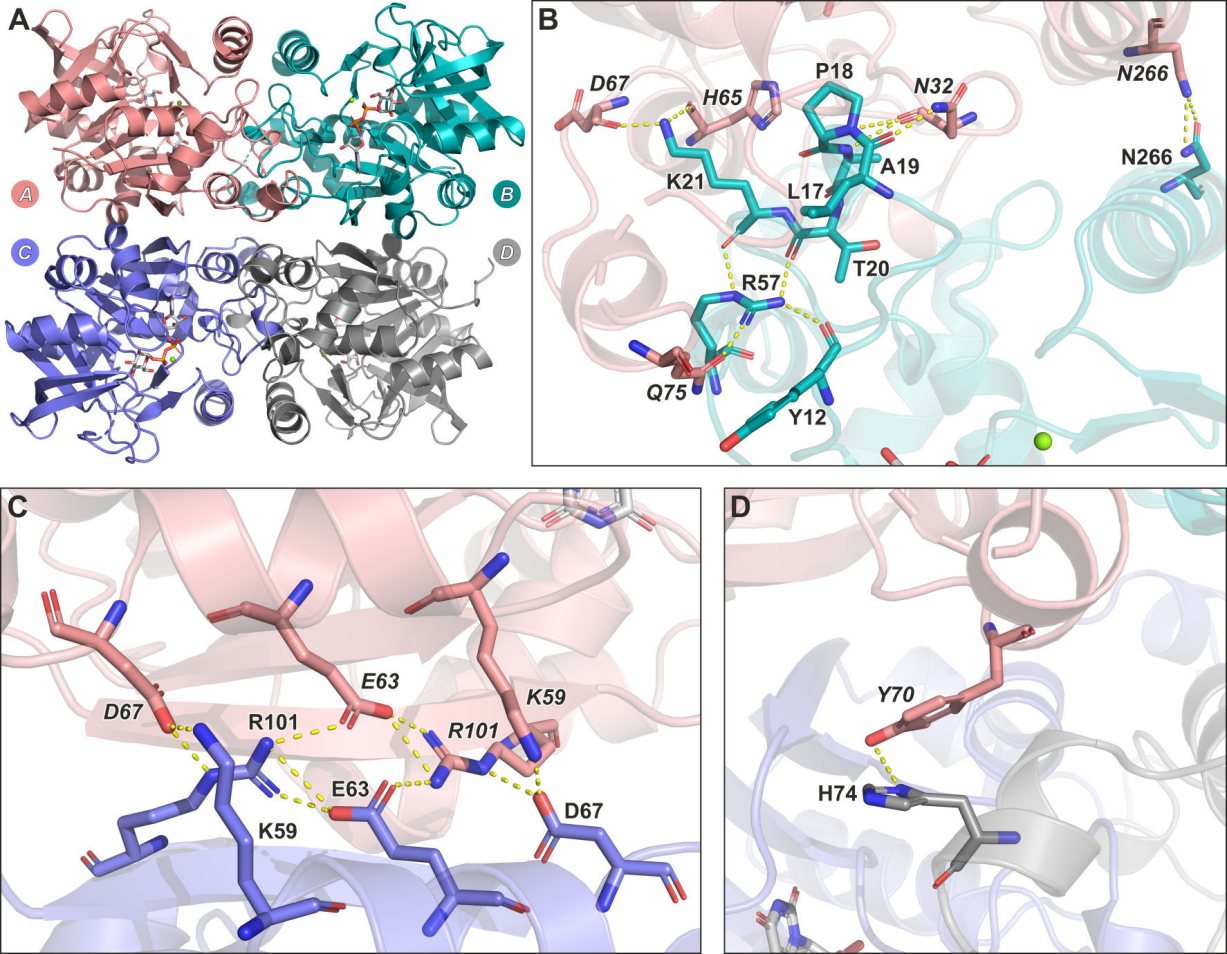
Quaternary structure and intermolecular interactions in PaUGP. (**A**) Cartoon representation of the reconstructed PaUGP tetramer in complex with the reaction product UDP-Glc. Subunits A, B, C, and D are shown in pink, cyan, purple, and gray, respectively, with A/B and C/D forming tight dimers and A/C and B/D forming loose dimers. (**B**) Interactions across the tight dimer interface, shown exemplarily between subunits A and B. (**C**) Interactions across the loose dimer interface, shown exemplarily between subunits A and C. (**D**) Interactions between diagonally opposed subunits A and D. Interacting residues and UDP-Glc are shown in stick representation with oxygen, nitrogen, and phosphorus shown in red, blue, and orange, respectively. Interactions are presented as yellow dotted lines.

Since the catalytic function of various UGPs has been linked to their respective oligomeric state, it is crucial to consider the quaternary assembly when analyzing PaUGP structure-function relationships. Using symmetry-related objects, we reconstructed the biologically active PaUGP homotetramer as seen in HpUGP, which can be described as a dimer of dimers, with subunits A/B as well as C/D forming “tight” dimers, and A/C as well as B/D forming “loose” dimers, respectively (Fig. 4A). Subunits A and B, as an example of the tight dimer, are packed with a burial of 2,005 Å^2^ of solvent-accessible surface. A network of strong interactions, involving 12 residues, was identified at the tight dimer interface (Fig. 4B). As in HpUGP (48), subunits A and C, as an example of the loose dimer, are less densely packed with a total buried accessible surface of 1,054 Å^2^; however, a tight hydrogen bond (H-bond) network is established between four charged amino acids (Fig. 4C). The loose dimer interaction results in an intermolecular extension of the central β-sheet as previously described for *Escherichia coli, Sphingomonas elodea,* and *Bacillus subtilis* UGPs (47, 51, 52). Finally, a single interaction between diagonally opposed subunits (e.g., A/D) was observed (Fig. 4D).

**Fig 5:**
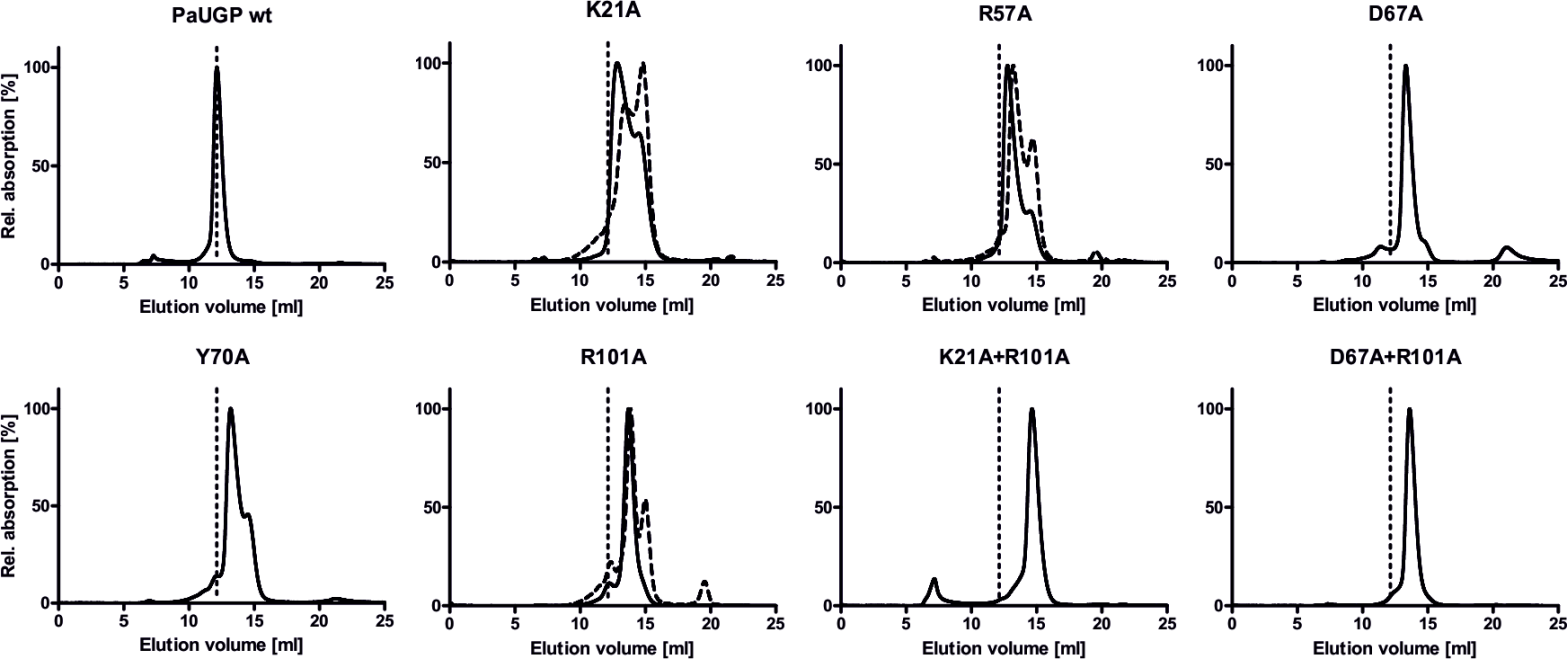
Size exclusion chromatography profiles of PaUGP WT and mutants demonstrating altered oligomerization. For uniform presentation, relative absorption is depicted on the *y*-axis, with the main peak of each protein set to 100% of peak intensity. For mutants that show dilution-dependent dissociation, the elution profile of the more diluted sample is shown as a broken line. The dashed vertical line indicates the elution volume of tetrameric WT PaUGP for reference.

**Fig 6:**
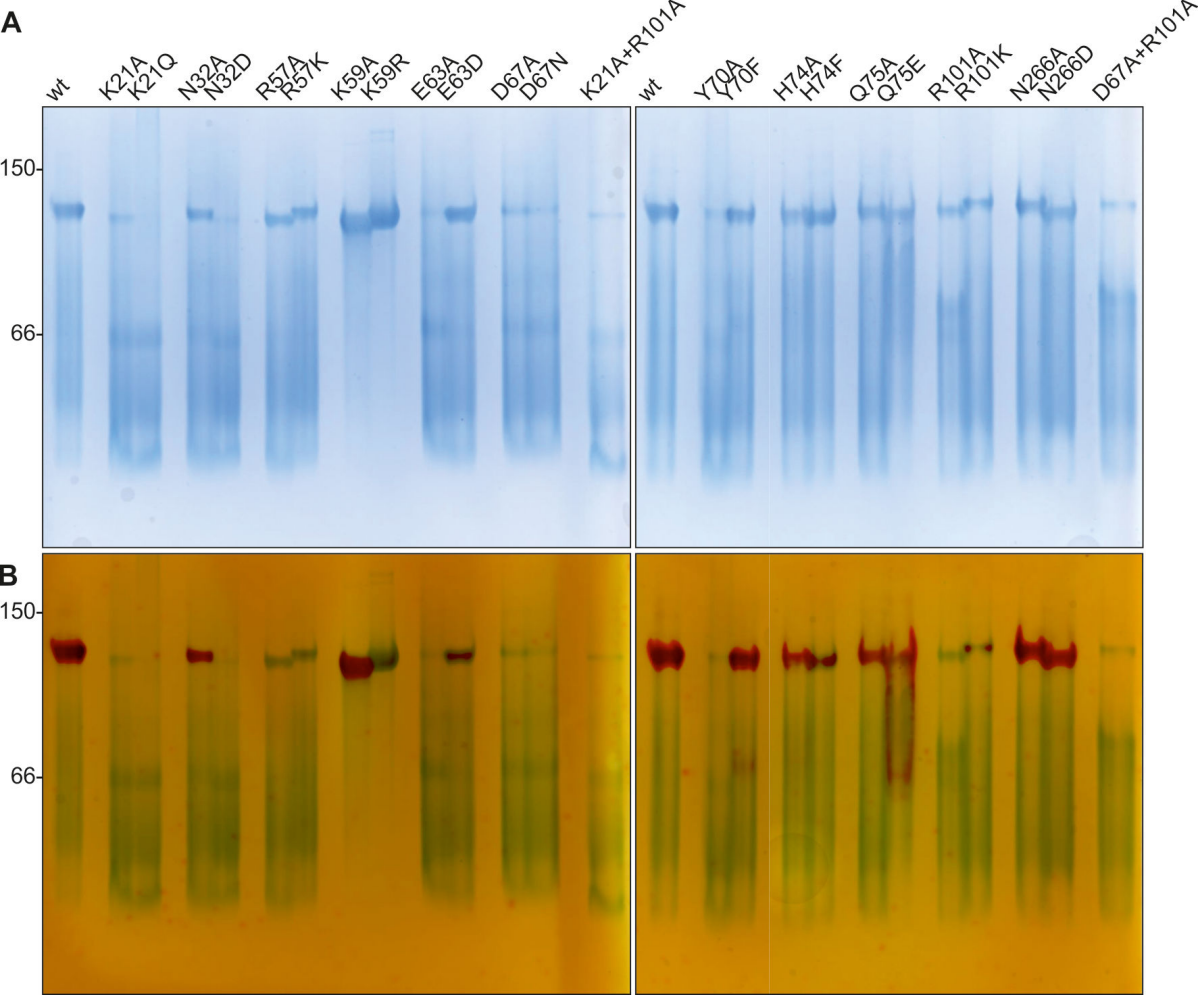
(**A**) Blue native-PAGE and (**B**) subsequent in-gel activity staining of WT and mutant PaUGP. A total of 13.5 µg of each protein was loaded on a uniform 12% native gel. Following electrophoresis, the same gel was incubated with the enzyme’s substrates (UTP and Glc-1-P), and the product PP_i_, produced by active UGP, was precipitated *in situ* with Ca^2+^ ions. The poorly documentable bands containing white CaPP_i_ precipitate were counter-stained with alizarin red for better visibility.

Out of 17 residues that appear to facilitate intermolecular interactions between subunits, 11 residues interact via their sidechains and were thus selected for mutagenesis (Fig. S8) and replaced both by alanine and conservatively. All mutants could be obtained as soluble proteins in purities comparable to WT PaUGP (Fig. S6B). The mutants’ oligomerization states and enzymatic activities were analyzed in solution (SEC and *in vitro* activity, Table 1; Fig. 5; Fig. S9 and S10) and in BN-PAGE followed by in-gel activity staining (Fig. 6), the latter serving as a non-quantitative method of visualizing the active UGP species. Of note, the elution behavior of proteins may differ between BN-PAGE and SEC due to the differences in buffer composition and pH. Furthermore, enzymatic activities visualized in-gel may differ from those measured via *in vitro* activity assay due to the drastically extended reaction time (4 min *in vitro* vs overnight incubation in-gel), with the latter allowing accumulation of product in protein bands containing even low UGP activity.

**TABLE 1:** Enzymatic activity and oligomeric state of PaUGP mutants *in vitro^a^*

Protein	*In vitro* activity (% of WT)	Elution volume(s) (mL)	Oligomeric state(s)
K21A^*b*^	5.00 ± 0.58	**14.79 ± 0.06**	**1.2**
		13.31 ± 0.15	2.3
K21Q^*b*^	1.26 ± 0.02	**14.45 ± 0.06**	**1.4**
		12.95 ± 0.00	2.7
N32A	56.16 ± 3.19	**11.90 ± 0.23**	**4.4**
		15.06 ± 0.11	1.1
N32D	109.79 ± 7.02	**11.74 ± 0.09**	**4.7**
		14.51 ± 0.08	1.4
R57A^*b*^	1.28 ± 0.03	**13.26 ± 0.05**	**2.4**
		14.72 ± 0.04	1.2
R57K^*b*^	2.36 ± 0.03	**12.60 ± 0.38**	**3.2**
		14.79 ± 0.05	1.2
K59A	2.31 ± 0.11	**12.36 ± 0.02**	**3.6**
K59R	1.66 ± 0.24	**12.11 ± 0.29**	**4.0**
E63A	7.60 ± 0.80	**12.31 ± 0.06**	**3.6**
E63D	5.68 ± 0.21	**12.14 ± 0.06**	**3.9**
		14.54 ± 0.05	1.3
D67A	3.31 ± 0.02	**13.33 ± 0.01**	**2.3**
		11.39 ± 0.02	5.5
D67N	2.54 ± 0.22	**13.58 ± 0.06**	**2.1**
		12.18 ± 0.00	3.8
Y70A	4.08 ± 0.12	**13.18 ± 0.01**	**2.5**
		14.52 ± 0.01	1.3
		12.24 ± 0.16	3.8
Y70F	47.16 ± 2.28	**12.27 ± 0.13**	**3.7**
		14.62 ± 0.00	1.3
H74A	60.32 ± 1.15	**12.28 ± 0.04**	**3.7**
		14.66 ± 0.03	1.3
H74F	19.70 ± 0.75	**12.38 ± 0.00**	**3.5**
		14.93 ± 0.00	1.1
Q75A	46.41 ± 3.70	**11.98 ± 0.23**	**4.2**
		14.69 ± 0.04	1.2
Q75E	15.35 ± 1.14	**12.40 ± 0.13**	**3.5**
R101A^*b*^	0.83 ± 0.03	**13.88 ± 0.01**	**1.8**
		14.85 ± 0.22	1.2
		12.33 ± 0.03	3.6
R101K^*b*^	1.31 ± 0.13	**13.48 ± 0.23**	**2.1**
		14.77 ± 0.17	1.2
N266A	64.89 ± 3.12	**12.11 ± 0.39**	**4.0**
		14.69 ± 0.10	1.2
N266D	104.86 ± 5.67	**11.96 ± 0.25**	**4.2**
		14.71 ± 0.00	1.2
K21A + R101A	0.32 ± 0.02	**14.62 ± 0.05**	**1.3**
D67A + R101A	1.57 ± 0.06	**13.64 ± 0.10**	**2.0**

^*a*^ *In vitro* activity (forward reaction) was quantified using the EnzChek assay. The calculated specific activities are given as means ± SEM of at least three individual experiments, each performed in technical triplicate, and expressed as a percentage of WT activity, which was defined as 100%. SEC elution volumes of distinguishable peaks are given as means ± SD of at least two individual experiments. Oligomerization states were calculated based on elution volumes of proteins of known size. Where multiple protein peaks were observed, the elution volume and calculated oligomeric state of the main peak are given in bold.

^*b*^ Mutants K21A/R, R57A/K, and R101A/K showed dilution-dependent behavior; elution volumes and oligomerization states given here refer to the more diluted sample (compare Fig. 5 and Fig. S9, broken lines).

#### Interactions across the tight dimer interface

Multiple contacts are established between subunits engaging in tight dimer formation (Fig. 4B), specifically between residues in α1, α4, α5, β3, and adjacent loops (Fig. S5A and S8). These include interactions with the A10-K21 sequence that borders the active site and contains the highly conserved GXGTRXLPXTK motif (Fig. S8), including G11 and R15, which are crucial for PaUGP activity (Fig. S5C).

N266, which is not conserved across bacterial UGPs (Fig. S8), interacts with its counterpart. Mutants N266A and N266D were mostly tetrameric with a small monomeric fraction in solution (Fig. S9). N266D retained full WT activity in solution, while N266A was reduced to ca. 65% of WT activity (Table 1). In BN-PAGE/activity staining, both mutants migrated predominantly as tetramers with strong activity staining (Fig. 6). These findings suggest a minor role of N266 in PaUGP oligomerization and activity.

N32 forms H-bonds to the neighboring subunit’s P18 (backbone) and A19 (backbone) via its backbone and to the backbone of L17 via its sidechain. PaUGP N32A and N32D mutants eluted mostly as tetramers in SEC, with minor monomer peaks (Fig. S9). N32A was reduced to ca. 56% of WT activity in solution, while N32D displayed full activity (Table 1), in line with the fact that the corresponding position is occupied by aspartic acid in other bacterial UGPs (Fig. S8). In contrast, in BN-PAGE, N32A displayed a clear, enzymatically active tetramer band, whereas N32D was mostly dissociated and displayed no activity staining (Fig. 6), which provides a first hint that tetramer formation is a prerequisite for PaUGP activity.

R57 and Q75 form H-bonds across the tight dimer interface. Q75A was mostly tetrameric in SEC and retained ca. 46% of WT activity, whereas Q75E was only 15% active and displayed a slightly shifted peak in SEC (calculated status 3.5, Table 1; Fig. S9), possibly indicating partial dissociation into dimers due to charge repulsion. Similarly, Q75A primarily migrated as an enzymatically active tetramer in BN-PAGE, whereas Q75E uniquely migrated as a smear between tetramer and dimer bands, which likewise contained active UGP (Fig. 6). These findings suggest that Q75 is negligible for tetramer stability, but uncharged amino acids are favored in this position. Mutants of R57, however, showed dilution-dependent dissociation in SEC, with R57A eluting as dimer and monomer and R57K as an apparent trimer (likely representing a tetramer/dimer equilibrium) and monomer (Table 1; Fig. 5; Fig. S9). Both mutants were almost inactive in solution (Table 1) and inactive in BN-PAGE, although tetramer bands were clearly visible (Fig. 6). Likely, mutation of R57 affects both PaUGP tetramer stability and activity due to its strong intramolecular H-bond network with the backbones of Y12, T20, and K21 (Fig. 4B), which are located within the highly conserved A10-K21 active site loop (Fig. S8). Similarly, conformational changes imposed by the mutation of R57’s interaction partner Q75 might impact PaUGP activity by altering the conformation and/or mobility of the A10-K21 loop. In HpUGP (PDB 3JUK), both R57 and Q75 are conserved; however, no interaction could be observed in the crystal structure (48) as the sidechains of residues E71-T79 are not resolved (Fig. S8), indicating that this region is flexible and may adopt multiple conformations.

The sidechain of strictly conserved K21 interacts with the backbones of H65 and D67; these interactions are strictly conserved across all crystallized bacterial UGPs (Fig. S11). K21A and K21Q exhibited ca. 5% and 1% residual activity, respectively, and eluted as dimer/monomer mixtures with a concentration-dependent ratio in SEC (Table 1; Fig. 5; Fig. S9). Interestingly, *in vitro* activity curves of both K21 mutants were nonlinear (Fig. S10B). Together, these findings suggest that K21 mutants can still form some loose dimer but are prone to further dissociation—coupled to progressive loss of activity—upon dilution, as occurs in the *in vitro* activity assay. In BN-PAGE, both K21 mutants eluted mainly as dimers and monomers, with a faint tetramer band in K21A but no in-gel activity visible for either mutant (Fig. 6).

#### Interactions across the loose dimer interface

The sidechain of D67 interacts with those of K59 and R101, the latter of which also engages in both inter- and intramolecular H-bonds with sidechains of E63 (Fig. 4C). K59A and K59R migrated like WT in SEC (Fig. S9) and as extremely stable tetramers in BN-PAGE (Fig. 6). Although both mutants displayed drastically diminished *in vitro* activity (Table 1), suggesting a functional role of the K59/D67 interaction, particularly K59A displayed clear in-gel activity staining (Fig. 6). This is likely due to the high tetramer stability of these mutants in BN-PAGE, combined with the drastically extended in-gel reaction time, which allows product accumulation even in bands containing low UGP activity. E63A and E63D remained almost entirely tetrameric in solution (Table 1; Fig. S9) but were less than 8% and 6% active, respectively (Table 1). In BN-PAGE, E63A was fully dissociated and showed no activity, while E63D displayed a clear tetramer band with some UGP activity (Fig. 6). These results indicate that E63 plays an important functional role in PaUGP activity via its inter- and/or intramolecular interactions with R101 (which could be partially maintained by the E63D mutation) and that the observed strong ionic interaction network contributes to tetramer stability.

Mutants of R101 dissociated into dimers and (at higher dilution) monomers in SEC and lost nearly all activity (Table 1; Fig. 5; Fig. S9), indicating R101’s importance for both oligomerization and activity. In BN-PAGE, R101 mutants were partially dissociated, with faint activity staining only in an R101K tetramer band (Fig. 6). Combination of mutations R101A and K21A (the latter of which disturbs primarily tight dimer formation) resulted in an additive effect, yielding an inactive monomeric enzyme (Table 1; Fig. 5 and 6; Fig. S9) and underlining that R101 plays a key role in loose dimer formation.

Mutation of D67 to alanine or asparagine caused a shift to a dimeric species in SEC and a nearly complete loss of *in vitro* activity (Table 1; Fig. 5; Fig. S9). Likewise, both D67A and D67N were mostly dissociated and inactive in BN-PAGE (Fig. 6). Although D67 also participates in tight dimer formation via its backbone, replacement of its sidechain should primarily interrupt loose dimer formation. Indeed, a D67A + R101A double mutant eluted similar to D67 and R101 single mutants in SEC (Table 1; Fig. 5; Fig. S9) and BN-PAGE (Fig. 6), underlining that both residues facilitate loose dimer formation.

#### “*Crossover*” interaction

The sidechain of Y70, uniquely, could form H-bonds with H74 of the subunit located diagonally across (i.e., subunits A/D, Fig. 4D). H74F and H74A mutants were mostly tetrameric and partially active in solution (20% and 60% of WT, respectively; Table 1; Fig. S9) and migrated mostly as partially active tetramers in BN-PAGE (Fig. 6). Y70A dissociated into dimers and monomers, accompanied by a drop to 4% of WT activity (Table 1; Fig. 5), whereas Y70F, which could retain some hydrophobic interactions with H74, was stably tetrameric and partially active in solution (47% of WT; Table 1; Fig. S9) and BN-PAGE (Fig. 6).

To summarize the tetramer analysis, out of 11 mutated residues whose sidechains engage in contacts between PaUGP subunits, K21, D67, and R101 were the most crucial for oligomer stability, as even conservative mutation led to complete dissociation of the native PaUGP tetramer, which was accompanied by nearly complete loss of activity. Because K21 and R101 are located in the vicinity of essential active site residues (compare Fig. S8), we hypothesize that they facilitate important intermolecular interactions stabilizing the active site, which are abolished upon mutation. However, it cannot be excluded that these mutations also influence PaUGP function via local conformational changes. D67, in contrast, provides interactions across both dimeric interfaces but is remote from the catalytic center. Therefore, its mutation most likely affects PaUGP activity solely via interference with tetramerization, i.e., in an exclusively allosteric manner. Moreover, even mutation of intermolecular contact residues that were not critical for tetramer stability caused a pronounced loss of activity (e.g., K59 and E63, Table 1), suggesting that also these residues engage in functional interactions enabled by the tetrameric assembly. Thus, we conclude that, although all residues forming the active site are contained within one subunit, intermolecular contacts of active site elements—enabled by the native tetrameric state—are crucial for PaUGP activity.

### Host-pathogen comparisons and implications for selective inhibition

We have previously studied structure-function relationships of UGPs from human (HsUGP) and the protozoan parasite *Leishmania major* (LmUGP) in detail (29, 36, 53). Comparison of the active site architecture of PaUGP with both eukaryotic UGPs (Fig. S12A and C through E) reveals striking similarities, and several key active site residues are conserved strictly or functionally, despite the lack of conservation on amino acid sequence level between bacterial and eukaryotic UGPs. Similar structural conservation of the active site exists, e.g., between plant UGP and protozoan UDP-sugar pyrophosphorylase, despite less than 20% identity between the proteins; consequently, several promising inhibitors identified by library screening against either enzyme turned out to inhibit both pyrophosphorylases (54). These observations strongly suggest that inhibitors binding to the PaUGP active site would cross-react with HsUGP, underlining the necessity of targeting unique structural elements linked to enzymatic activity in order to achieve selective inhibition of the bacterial UGP.

**Fig 7:**
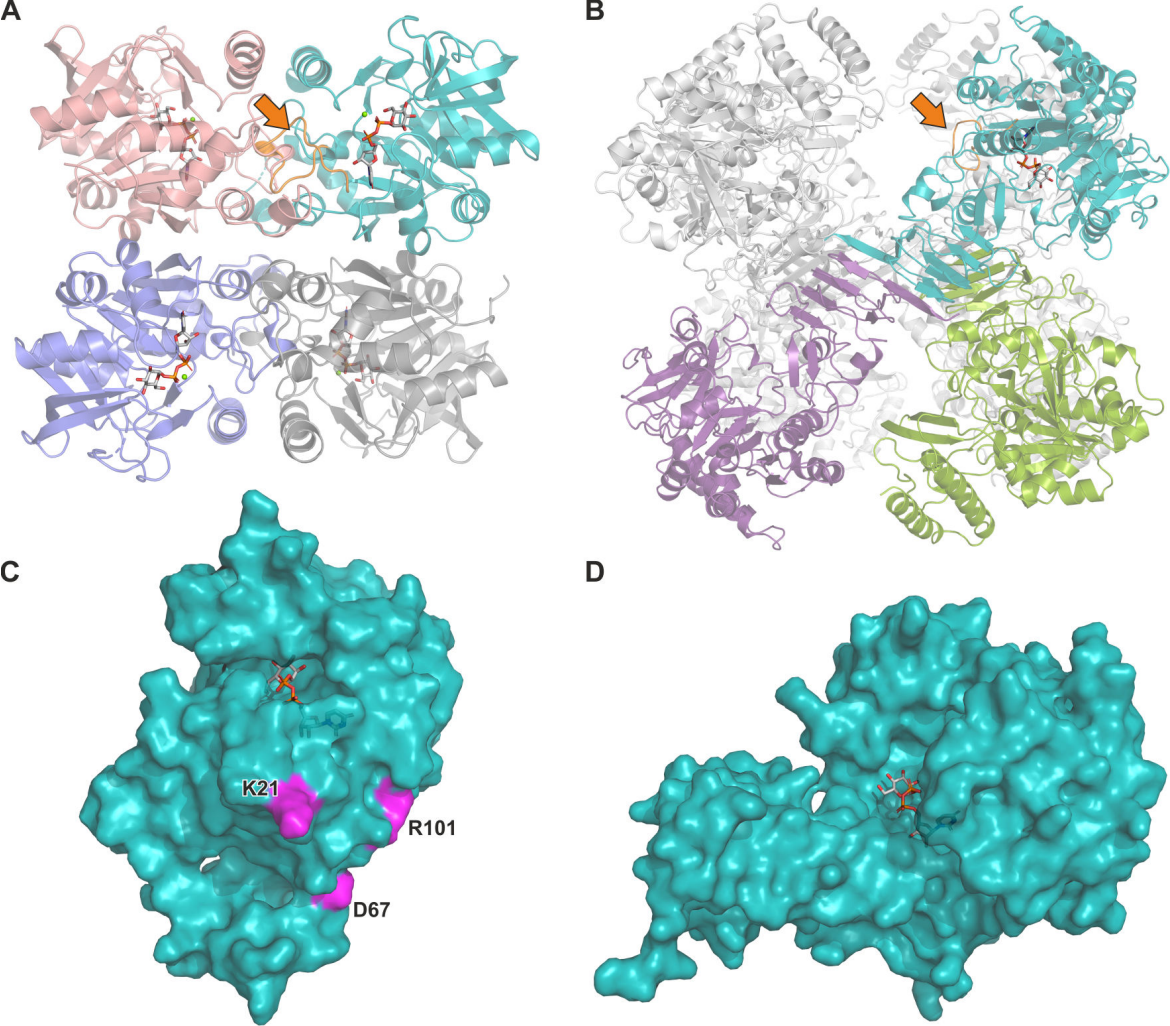
Quaternary and tertiary structure comparison of PaUGP (this work) and HsUGP (PDB 4R7P). (**A**) Cartoon representation of tetrameric PaUGP, with tight dimers formed between pink/cyan and purple/gray subunits, and loose dimers formed between pink/purple and cyan/gray subunits, respectively. (**B**) Cartoon representation of octameric *Homo sapiens* UGP in complex with UDP-Glc with the oligomeric assembly representatively shown for the cyan and purple subunits (end-to-end dimer) and cyan and green subunits (side-by-side dimer). The remaining five subunits are depicted in gray for clarity. In the respective cyan subunit, the PaUGP active site loop A10-K21 and its structural homolog in HsUGP are highlighted in orange (see the arrow). (**C**) Surface representation of a PaUGP subunit in complex with UDP-Glc, with key residues K21, D67, and R101 highlighted in pink. (**D**) Surface representation of a HsUGP subunit in complex with UDP-Glc, shown in the same orientation as PaUGP in panel C. In all panels, UDP-Glc is shown as gray sticks, with phosphorus, oxygen, and nitrogen atoms colored orange, red, and blue, respectively.

Our previous work has shown that, similar to PaUGP, HsUGP enzymatic activity depends on oligomerization, which enables a functionally crucial intermolecular interaction stabilizing the active site (29, 36). However, in HsUGP, this is facilitated by octamerization via a C-terminal β-helix domain not present in PaUGP (compare Fig. 7A and B); *vice versa*, none of the PaUGP key oligomerization residues are conserved in HsUGP (compare Fig. 7C and D). These important differences in the two enzymes’ relationships between quaternary structure and enzymatic function suggest that targeting the functionally crucial PaUGP tetramerization interfaces, which are absent in the human enzyme, provides an opportunity for selectively inhibiting PaUGP.

## DISCUSSION

### Role of UGP in *P. aeruginosa in vitro* and *ex vivo* virulence

Bacterial UGPs have been suggested as promising “anti-virulence” drug targets due to their central role in UDP-sugar metabolism and virulence factor synthesis (1). Priebe and coworkers (6) showed that *P. aeruginosa galU*^−^ strains display attenuated virulence in murine *in vivo* corneal and acute pneumonia infection models, which was attributed to increased bacterial clearance by the immune system. Considering that these immune mechanisms are not at play in a cell culture setting, the results of our Calu-3 infection experiments (compare Fig. 1B) suggest that UGP-deficient PAO1 exhibits significantly reduced inherent cytotoxicity compared to WT, whereas Priebe et al. (6) found no significant differences between *in vitro* cytotoxicity of PAO1 WT and a *galU*^−^ mutant strain. The discrepancy between both studies may be caused by the use of different cell lines (16HBE14o vs Calu-3) and/or different methods and time points of cytotoxicity quantification (LDH assay 3 h post-infection vs calcein assay 24 h post-infection).

To confirm the importance of UGP for *P. aeruginosa* virulence and further support the enzyme’s suggested use as a drug target, we moved into the more complex and human-relevant organotypic *ex vivo* model of human PCLS (Fig. 1C and D), which represent snippets of intact lung tissue morphology. Our study, to the best of our knowledge, represents the first report of a *P. aeruginosa ex vivo* infection model using native human lung tissue providing data on bacterial fitness and virulence as well as host tissue viability and immune readouts. Here, we show that PAO1 colonizes human PCLS, induces a pro-inflammatory response, and ultimately leads to tissue death, with PAO1 *galU^−^* exhibiting significantly reduced cytotoxicity compared to WT. Levels of cytokines secreted by PCLS were highly variable, which is likely due to baseline differences in the immunological response between the five individual tissue donors that may be influenced by a variety of factors including age, sex, genetics, comorbidities, and infection history.

While the mechanistic link between UGP function and cytotoxicity remains unknown, we hypothesize that the LPS core truncation of PAO1 *galU*^−^ could indirectly influence the efficiency of *P. aeruginosa’s* secretion systems, particularly type III and VI (T3SS and T6SS), which directly inject toxic effector proteins into host cells (55). T3SS has been linked to disease severity in patients with pneumonia and to *P. aeruginosa* pathogenesis in murine lung and corneal infection models (6, 56, 57) and is thus considered the most relevant for virulence toward host cells. Importantly, attachment to the host cell needs to be established for the delivery of PAO1’s cytotoxic exoproteins exoT, exoS, and exoY. LPS is involved in the adhesion of *P. aeruginosa* to surfaces and host receptors; specifically, epithelial cell binding and ingestion of bacteria are mediated by the CFTR, which recognizes the glucose-rich outer core oligosaccharide of LPS (58). Zaidi et al. (59) further reported that association and entry of *P. aeruginosa* into corneal cells depend on an exposed terminal Glc residue in the LPS outer core, which is absent in UGP-deficient strains (18). Accordingly, altered binding to cell surface receptors and thus inability of the PAO1 *galU^−^* mutant to effectively deliver its exotoxins into host cells could contribute to the reduced cytotoxicity observed in our infection models. Infection with PAO1 WT furthermore triggered significantly higher TNF-α and IL-6 secretion in Calu-3 cells than the *galU^−^* mutant, which might similarly be attributed to the altered receptor-mediated binding of *galU^−^* bacteria to epithelial cells, leading to an overall reduction of immunogenic stimulation of host cells. Further studies, which were beyond the scope of this work, are required to confirm these hypotheses.

We furthermore observed changes in pyocyanin production as well as swimming and swarming motility between WT and *galU^−^*, all of which may affect *P. aeruginosa* virulence (see Supplemental Discussion).

### PaUGP oligomerization and selective inhibition potential

In contrast to their overall conserved active site architecture, UGPs from different kingdoms of life utilize vastly different enzymatic mechanisms linked to their diverse quaternary structures, e.g., whereas human UGP forms functional octamers (29) to enable intermolecular interactions stabilizing the active site geometry (36), monomeric *L. major* UGP achieves a similar stabilizing interaction in an intramolecular fashion, utilizing high conformational mobility (53).

While the majority of bacterial UGPs characterized to date are tetramers, it was previously unknown whether these represent the active oligomeric species, or whether dissociation into active monomers occurs, as described for plant UGPs (37). Here, we show that recombinant PaUGP is an enzymatically active tetramer in solution and BN-PAGE, and present the crystal structure which allowed the reconstruction of a functional tetramer established by “tight” and “loose” dimer interactions as reported for several other bacterial UGPs (47–51). To dissect the relationship between PaUGP activity and oligomerization, we mutated 11 amino acids whose sidechains appear to be involved in interactions between subunits and analyzed their oligomerization state and activity (Table 1). All mutants retaining full or considerable (≥50% of WT) UGP activity were tetrameric, whereas all mutations that triggered dissociation caused nearly complete loss of activity, underlining that tetramers are the active oligomeric species of PaUGP. Seeking a mechanistic link between PaUGP tetramerization and activity, we identified two regions in which intermolecular contacts likely impact catalytic function: (i) the loop encompassing active site residues Q102-G107 is adjacent to R101, which interacts with E63 and D67 across the loose dimer interface; and (ii) the highly conserved A10-K21 active site loop, via residues L17-K21, interacts with N32, H65, and D67 across the tight dimer interface (Fig. 4; Fig. S8). We therefore hypothesize that intermolecular interactions established in the PaUGP tetramer enable enzymatic activity by stabilizing the active site architecture. This is conceptually similar to the mechanism of the human enzyme, although HsUGP octamerization facilitates stabilization of the sugar-binding loop, while PaUGP tetramerization involves the nucleotide-binding region of the active site, which in HsUGP does not interact with neighboring subunits (compare Fig. 7A and B, orange arrow, and Fig. S11L). Importantly, both enzymes employ entirely different modes of oligomerization via structural elements that, unlike the catalytic center, are non-conserved (compare Fig. 7). In particular, the identified PaUGP key residues K21, D67, and R101, whose mutation disrupted both oligomerization and activity, are not present in HsUGP, suggesting that targeting PaUGP tetramerization interfaces may enable selective inhibition of the bacterial enzyme. Interestingly, our analysis of all crystallized bacterial UGPs revealed that the interactions of K21 with D67 and adjacent residues at the tight dimer interface are strictly conserved (Fig. S11A through K). Therefore, blocking the intermolecular interactions, particularly around D67—which, due to its remoteness from the active site, would interrupt tetramerization and consequently abolish enzymatic activity in an allosteric manner—could represent a novel approach to selectively inhibiting not only PaUGP but also, due to the conservation of these interactions, bacterial UGPs in general. Attractive target pathogens are, for example, *S. pneumoniae* and *K. pneumoniae*, both of which absolutely require UGP for CPS synthesis.

In summary, our data demonstrate that PaUGP is an attractive drug target due to its importance for virulence toward human lung cells and tissue. The use of human PCLS as an organotypic *ex vivo* model of the human lung emphasizes the relevance of these results. We demonstrate that native tetramerization is essential for PaUGP activity and show that UGPs of the pathogen and its host utilize different structural and mechanistic features in the context of their respective quaternary assembly to achieve catalytic function. In conclusion, our findings suggest the possibility of targeting unique oligomerization interfaces of PaUGP—and potentially bacterial UGPs in general—to disrupt the active oligomeric species and selectively inhibit the bacterial enzyme.

## MATERIALS AND METHODS

### Cell culture of human lung epithelial cells

The Calu-3 human lung adenocarcinoma cell line (ATCC-HTB-55) was maintained in continuous culture with Dulbecco’s Modified Eagle’s Medium (DMEM) (Gibco). The culture medium was routinely supplemented with 10% heat-inactivated fetal bovine serum, 50 ng/mL gentamycin, 2 mM glutamine, and 0.3% phenol red. For infection experiments, cells were seeded (4 × 10^5^ cells/well) into 12-well plates. For pyocyanin treatment experiments, cells were seeded into 48-well plates. Cells were maintained at 37°C in 5% CO_2_ until they were at least 80% confluent, at which time they were utilized in the desired experiments.

### Preparation and culture of *ex vivo* human lung tissue

Precision-cut lung slices were prepared as previously described (60). Human lung lobes were obtained from male and female patients who underwent lung tumor resection. Tumor-free tissue was processed immediately on the day of resection. Briefly, the bronchi were cannulated, and the lungs were inflated with 2% low-gelling agarose in 37°C warm serum-free DMEM/nutrient mixture F-12 Ham (DMEM/F12, pH 7.2–7.4) with L-glutamine and 15 mM HEPES [4-(2-hydroxyethyl)-1-piperazineethanesulfonic acid] without phenol red from Gibco (Thermo). After polymerization of agarose on ice, tissue cores were cut into 200–300 µm thick slices using a Krumdieck tissue slicer (Alabama Research and Development) filled with 4°C cold Earle’s Balanced Salt Solution (Sigma-Aldrich). Subsequently, PCLS were incubated in DMEM/F12, pH 7.2–7.4 with L-glutamine and 15 mM HEPES without phenol red from Gibco (Thermo). PCLS were maintained for less than 24 h under standard cell culture conditions (37°C, 5% CO_2_, and 100% humidity) before infection.

### Bacterial culture and growth kinetics

*P. aeruginosa* PAO1 (DSM 19880) was obtained from the German Collection of Microorganisms and Cell Cultures (Braunschweig, Germany). A UGP-deficient PAO1 mutant strain (PW4507), carrying a transposon inserted into the *galU* gene after base 39, was obtained from the *P. aeruginosa* PAO1 Two-Allele Transposon Mutant Library (44) (https://www.gs.washington.edu/labs/manoil/libraryindex.htm). Transposon insertion into the *galU* gene was confirmed by PCR and sequencing.

Overnight cultures of PAO1 WT and UGP-deficient PAO1 (*galU^−^)* were grown in lysogeny broth for 16 h at 37°C, 150 rpm on a shaking incubator. For the determination of *in vitro* growth kinetics, overnight cultures were diluted to an OD_600_ of 0.002 in LB, DMEM, or DMEM/F12, and 100 µL of bacterial suspension was added to 100 µL of growth medium in a flat bottom 96-well plate (TPP). OD_630_ was measured in technical triplicates for 24 h at 37°C every 30 min with intermittent shaking in a plate reader (Tecan).

### Infection of cells and *ex vivo* lung tissue with *P. aeruginosa*

PAO1 WT or *galU*^−^ overnight cultures were diluted to an OD_600_ of 0.002. Calu-3 cells were infected by adding 2 × 10^5^ colony-forming units (CFU) to each well of a 12-well plate and centrifuging at 300 × *g* for 5 min to increase the contact between bacteria and cells. PCLS were infected by adding 1 × 10^5^ CFU to each well of a 24-well plate. Infected cells and PCLS were incubated for 1 h at 37°C, 5% CO_2_, after which the inoculum was removed, cells/PCLS were washed with phosphate-buffered saline (PBS), and supplied with fresh medium. Six hours after infection, cell and tissue culture supernatants were collected and frozen at -80°C for the quantification of pro-inflammatory cytokines, and the wells were filled with fresh medium.

### Determination of cell and tissue viability

The viability of Calu-3 cells and PCLS was measured 24 h post-infection. Calcein AM (Thermo Fisher) was reconstituted according to the manufacturer’s manual and diluted in PBS to a final concentration of 80 µM. After the removal of the culture medium, calcein AM staining solution was added to each well of cultured cells or PCLS and incubated for 45 min at 37°C, 150 rpm on a shaking incubator. After the removal of the staining solution and washing with PBS, cells and PCLS were lysed in 1% Triton-X in PBS (Sigma). Lysates were transferred to a black 96-well plate, and fluorescence at 485 nm (excitation)/535 nm (emission) was measured in a plate reader (Tecan). Fluorescence was recorded as relative fluorescence units, and the uninfected controls were defined as having 100% viability.

### Determination of bacterial loads

Cell and tissue culture supernatants and lysates were serially diluted in PBS-Tween (0.05%) and plated on LB agar. The plates were incubated at 37°C, and the number of CFU was counted on the following day.

### Quantification of pro-inflammatory cytokines

Secretion of the cytokines IFN-γ, IL-1β, IL-2, IL-4, IL-6, IL-8, IL-10, IL-12p70, IL-13, and TNF-α by Calu-3 cells and PCLS was measured by multiplex ELISA using the V-PLEX Proinflammatory Panel 1 Human Kit (Meso Scale Discovery) according to the manufacturer’s instructions.

### Quantification of pyocyanin and pyocyanin treatment of Calu-3 cells

Pyocyanin content in sterile filtrated overnight cultures and culture supernatants of PCLS and Calu-3 cells was determined by measuring the absorption at 318 nm (61) using a UV-vis spectrophotometer (Thermo). To normalize pyocyanin content to bacterial load, absorption at 318 nm was divided by the respective sample’s OD_600_ value in the case of overnight cultures or by (log) CFU counts in the case of PCLS and Calu-3 supernatants. Purified pyocyanin (Sigma) dissolved in dimethylformamide and diluted in DMEM was used to generate a standard curve and calculate pyocyanin produced by *P. aeruginosa*. Calu-3 cells were cultured in 48-well plates as described above and treated with different pyocyanin concentrations for 24 h. Cell viability was determined by calcein fluorescence as described above (see “Determination of cell and tissue viability”).

### Bacterial motility

Motility assessment was modified from Marr et al*.* (62). Swimming motility was evaluated on BM2 plates [40 mM K_2_HPO_4_, 22 mM KH_2_PO_4_, 7 mM (NH_4_)_2_SO_4_, 2 mM MgSO_4_, 10 µM FeSO_4_, and 0.4% (wt/vol) glucose] containing 0.3% agar and swarming on modified BM2 [with 0.1% (wt/vol) casamino acids instead of (NH_4_)_2_SO_4_] plates containing 0.5% agar. Three 1 µL spots of PAO1 overnight cultures were pipetted on the BM2 agar plate and incubated overnight at 37°C. On PAO1 *galU*^−^ plates, an additional reference spot of PAO1 WT was added to the center of the plate to account for possible differences in agar composition between individual plates. All motility assays were performed with triplicate agar plates. The diameters of individual swimming zones on each plate were manually measured and averaged.

### Generation of a PaUGP expression construct

We amplified the *P. aeruginosa galU* gene, encoding the UGP enzyme, from plasmid PaCD00006903 obtained from the Plasmid Repository of the Arizona State University (https://dnasu.org/). The PCR product was digested via BamHI and XhoI restriction sites introduced via primers and ligated into a likewise digested, previously modified pET22b expression vector (Novagen) containing an N-terminal Strep-tagII followed by a thrombin cleavage site (sequence: MASWSHPQFEKGALVPRGS), yielding the construct Strep-PaUGP.

### Recombinant expression of Strep-PaUGP

Plasmids were transformed into Ca^2+^-competent *E. coli* BL21(DE3) by heat shock. Recombinant expression was carried out using the Enpresso B500 Kit (Enpresso) in a culture volume of 100 mL, but otherwise according to the manufacturer’s instructions. Isopropyl-1-thio-β-D-galactopyranoside at 1 mM concentration served as an induction agent. Bacteria were harvested by centrifugation (4,000 × *g*, 15 min, 4°C) and washed twice with PBS.

### Point mutations

Site-directed mutagenesis was performed according to Liu and Naismith (63) using primers listed in Table S3. The plasmid Strep-PaUGP encoding the wild-type enzyme served as a PCR template. Parental, methylated DNA was digested with DpnI after PCR. The integrity of all plasmids was confirmed by sequencing (Eurofins Genomics).

### Purification of Strep-tagged PaUGP by affinity chromatography

Expression culture pellets were resuspended in 25 mL of buffer W (100 mM Tris-HCl pH 8.0 and 150 mM NaCl) with protease inhibitors (cOmplete ULTRA Tablets, Roche). Bacterial lysis was performed by sonication with a Branson sonifier (50% amplitude, 10 cycles of 30 s alternating with 30 s of rest) while being cooled on ice. The suspension was centrifuged (20,000 × *g*, 30 min, 4°C), and the supernatant was passed through a 0.8 µL filter before loading the cleared lysate onto a 5 mL Strep-Tactin Sepharose column (IBA) equilibrated with buffer W. Unbound proteins were removed by washing with 10 column volumes of buffer W. Strep-tagged PaUGP was eluted with 100% buffer E (buffer W plus 2.5 mM desthiobiotin) on an ÄKTA system (Cytiva). Buffer exchange into 50 mM Tris-HCl pH 8.0, 100 mM NaCl, and 10 mM MgCl_2_ was performed using a HiPrep 26/10 desalting column (Cytiva), and aliquots were stored at −80°C after flash-freezing in liquid nitrogen. Protein concentrations were calculated from the absorbance at 280 nm, taking into account each protein’s individual extinction coefficient and molecular weight, which were calculated using ProtParam (http://web.expasy.org/protparam/).

### *In vitro* activity assay

*In vitro* activity of PaUGP was determined using the EnzChek pyrophosphate assay (Invitrogen) at 25°C in 100 µL volume in 96-well half-area flat-bottom microplates (Greiner Bio-One). Substrate concentrations were 1 mM UTP and Glc-1-P each. Reactions were initiated by adding 10 µL of recombinant PaUGP in suitable dilution to 90 µL of reaction mastermix. Product formation was continuously measured for 4 min at 360 nm in a Power-Wave TM 340 microplate reader (Bio-Tek). To correct the data for background activity caused by (pyro)phosphate contaminations, control measurements initiated with 10 µL of buffer were performed.

### SDS-PAGE analysis and immunoblotting

Protein integrity and purity of recombinant PaUGPs were assessed by SDS-PAGE according to Laemmli, using 12% SDS-polyacrylamide gels overlaid with a 5% stacking gel. Proteins were visualized by Coomassie staining. For Western blot analysis, proteins were transferred to nitrocellulose membrane (GE Healthcare), and Strep-tagged proteins were visualized with Strep-Tactin horse radish peroxidase conjugate (IBA) and the Pierce ECL Western Blotting Substrate kit (Thermo Scientific). Chemoluminescence was detected on an Amersham Imager 680 (GE Healthcare, software version 2.0.0).

### Size exclusion chromatography

Protein apparent molecular weights were determined by size exclusion chromatography using a Superdex 200 10/300 GL column (GE Healthcare) and a buffer composed of 50 mM Tris-HCl pH 8.0, 300 mM NaCl, and 10 mM MgCl_2_. For calibration, molecular weight marker proteins (Sigma) were subjected to chromatography under the same conditions.

### Blue native PAGE and in-gel activity staining

BN-PAGE was performed according to the protocol of Wittig et al. (64) for chromatographically purified proteins using a 12% polyacrylamide gel overlaid with a 3.5% stacking gel. A total of 13.5 µg of WT or mutant UGP was loaded. Alcohol dehydrogenase from yeast (150 kDa) and bovine serum albumin (66 kDa) were used as size standards. After documenting the Coomassie-stained protein bands on an Amersham Imager 680 (GE Healthcare), in-gel activity staining was performed at room temperature (RT) using a method modified from Manchenko (65). The gel was equilibrated in 50 mM glycine-KOH buffer pH 9.0 for 20 min and subsequently incubated overnight in 50 mM glycine-KOH buffer pH 9.0, 20 mM n-octyl β-D-glucoside, 8 mM MgCl_2_, 5.3 mM CaCl_2_, 2.3 mM UTP, and 1.3 mM Glc-1-P. Bands containing active UGP produce UDP-Glc and PP_i_, the latter forming a white precipitate in the presence of Ca^2+^ ions. The precipitate was counter-stained with alizarin red solution (Merck) for 1 h at RT, the gel was washed with 5% acetic acid/15% ethanol to minimize background staining and documented with an Amersham Imager 680 (GE Healthcare, software version 2.0.0).

### Protein crystallization of PaUGP in complex with UDP-Glc

Purified PaUGP protein (20 mg/mL final concentration) was pre-mixed with 10 mM of UDP-Glc for 1 h at 4°C before drops were set up with crystallization reservoir solution of 0.1 M sodium acetate trihydrate pH 4.6 and 2.0 M sodium formate using the sitting drop vapor diffusion method. Crystals were mounted and cryoprotected using a solution consisting of reservoir solution and 20% ethylene glycol. The crystals were flash frozen in liquid nitrogen at 100 K and sent to the Australian Synchrotron (Melbourne, Australia) for data collection.

### Data collection and crystal structure determination

X-ray diffraction data of single crystals were collected at the Australian Synchrotron MX2 beamline (66) using a wavelength of 0.9537 Å. A total of 3,600 images were collected on a Dectris EIGER 16M detector covering a rotation range of 360°. The detector distance was set to 270 mm with 36 s of total exposure and an oscillation range of 0.1° per image.

The collected data sets were processed using XDS (67) for indexing and integration and scaled using AIMLESS (68). Data quality was assessed using Phenix Xtriage (69), and molecular replacement was performed using Phaser (70) with search model PDB 3JUK. Phenix AutoBuild (71) was used to generate an initial model followed by manual inspection and adjustment in Coot (72) to further improve the model. Iterative refinement was achieved with Phenix and Coot. Data collection and refinement statistics are listed in Table S2. All structural analyses were done using Pymol (73). The crystal structure has been deposited into the Protein Data Bank (www.pdb.org) with the PDB code 8F73.

### Statistical evaluation

Data are presented as mean ± standard deviation. Statistical analysis was performed as indicated in each figure description (unpaired two-tailed *t*-test or one-way analysis of variance with Tukey’s multiple comparisons test) using GraphPad Prism version 9.5.0 for Windows (GraphPad Software, San Diego, CA, USA, www.graphpad.com). Statistical significance was denoted as *P* < 0.05 and is given as a numerical value in each graph.

## Data Availability

The crystal structure of PaUGP in complex with UDP-Glc has been deposited into the Protein Data Bank (PDB) with accession code 8F73.

## SUPPLEMENTAL MATERIAL

Supplemental material mbio.02114-23-s0001.docx Supplemental figures, tables, and discussion.

ASM does not own the copyrights to Supplemental Material that may be linked to, or accessed through, an article. The authors have granted ASM a non-exclusive, world-wide license to publish the Supplemental Material files. Please contact the corresponding author directly for reuse.
