# Supplementary material for: Tetramerization is essential for the enzymatic function of the Pseudomonas aeruginosa virulence factor UDP-glucose pyrophosphorylase
Source: mBio. 2024 Mar 12;15(4):e02114-23. doi: 10.1128/mbio.02114-23 (PMC11005391; doi:10.1128/mbio.02114-23)
Supplement: Supplemental material — Supplemental figures, tables, and discussion. [file mbio.02114-23-s0001.docx]

Supplemental material for

**Tetramerization is essential for enzymatic function of the *Pseudomonas aeruginosa* virulence factor UDP-glucose pyrophosphorylase**

Authors

Larissa Dirr^1*†^, Sven Cleeves^2†^, Isabel Ramón Roth^3^, Linghui Li^1‡^, Timm Fiebig^3^, Thomas Ve^1^, Susanne Häussler^4^, Armin Braun^2,5^, Mark von Itzstein^1^, Jana I. Führing^3*^

Affiliations

^1^ Institute for Glycomics, Gold Coast Campus, Griffith University, Gold Coast, 4222, Australia.

^2^ Fraunhofer Institute for Toxicology and Experimental Medicine ITEM, Member of Fraunhofer International Consortium for Anti-Infective Research (iCAIR), Member of the German Center for Lung Research (DZL), Biomedical Research in Endstage and Obstructive Lung Disease (BREATH), 30625 Hannover, Germany.

^3^ Institute of Clinical Biochemistry, Hannover Medical School, 30625 Hannover, Germany.

^4^ Department of Molecular Bacteriology, Helmholtz Centre for Infection Research, 38124 Braunschweig, Germany; Institute for Molecular Bacteriology, TWINCORE, Centre for Experimental and Clinical Infection Research, Hannover, Germany; Department of Clinical Microbiology, Copenhagen University Hospital - Rigshospitalet, 2100 Copenhagen, Denmark; Cluster of Excellence RESIST (EXC 2155), Hannover Medical School, Hannover, Germany.

^5^ Institute of Immunology, Hannover Medical School, 30625 Hannover, Germany.

^*^ Corresponding authors. Email: l.dirr@griffith.edu.au, fuehring.jana@mh-hannover.de

^†^ These authors contributed equally to this work.

^‡^ Present address: Shenzhen Longhua High School Education Group, 9 Huaxi road, Mingzhi street, Shenzhen, China, 518131.

Table of contents

[Supplemental figures 3](#_Toc156819671)

[Fig. S1 Growth kinetics of PAO1 WT and *galU^-^* over 24 h in LB medium, DMEM/F12 and DMEM 3](#_Toc156819672)

[Fig. S2 Pro-inflammatory cytokines in culture supernatants of Calu-3 cells and PCLS*.* 4](#_Toc156819673)

[Fig. S3 Different pyocyanin levels in PAO1 WT and *galU^-^* culture supernatants. 5](#_Toc156819674)

[Fig. S4 Analysis of PaUGP oligomerization state and identification of the enzyme via SDS-PAGE and Western Blot. 6](#_Toc156819675)

[Fig. S5 PaUGP subunit structure and mutational analysis of active site. 7](#_Toc156819676)

[Fig. S6 SDS-PAGE analysis of purified recombinant Strep-tagged wild-type and mutant PaUGP. 8](#_Toc156819677)

[Fig. S7 Oligomeric states and activities of PaUGP wt and active site mutants in solution 9](#_Toc156819678)

[Fig. S8 Multiple sequence alignment of bacterial UGPs. 11](#_Toc156819679)

[Fig. S9 Size exclusion chromatography profiles of PaUGP mutants of potential oligomerization-relevant residues 12](#_Toc156819680)

[Fig. S10 Activities of PaUGP mutants of potential oligomerization-relevant residues. 13](#_Toc156819681)

[Fig. S11 Conservation of intermolecular interactions across the tight dimer interface (pink and cyan subunits) and (if applicable) the loose dimer interface (pink and purple subunit) in bacterial UGPs. 15](#_Toc156819682)

[Fig. S12 Active sites of PaUGP, *Homo sapiens* UGP (HsUGP) and *Leishmania major* (LmUGP). 17](#_Toc156819683)

[Supplemental tables 18](#_Toc156819684)

[Table S1 Bacterial loads in cell/tissue lysates and culture supernatants. 18](#_Toc156819685)

[Table S2 Crystallographic statistics of PaUGP in complex with UDP-Glc 19](#_Toc156819686)

[Table S3 Mutagenesis primers and their sequences 20](#_Toc156819687)

[Supplemental discussion 22](#_Toc156819688)

[References 24](#_Toc156819689)

Supplemental figures


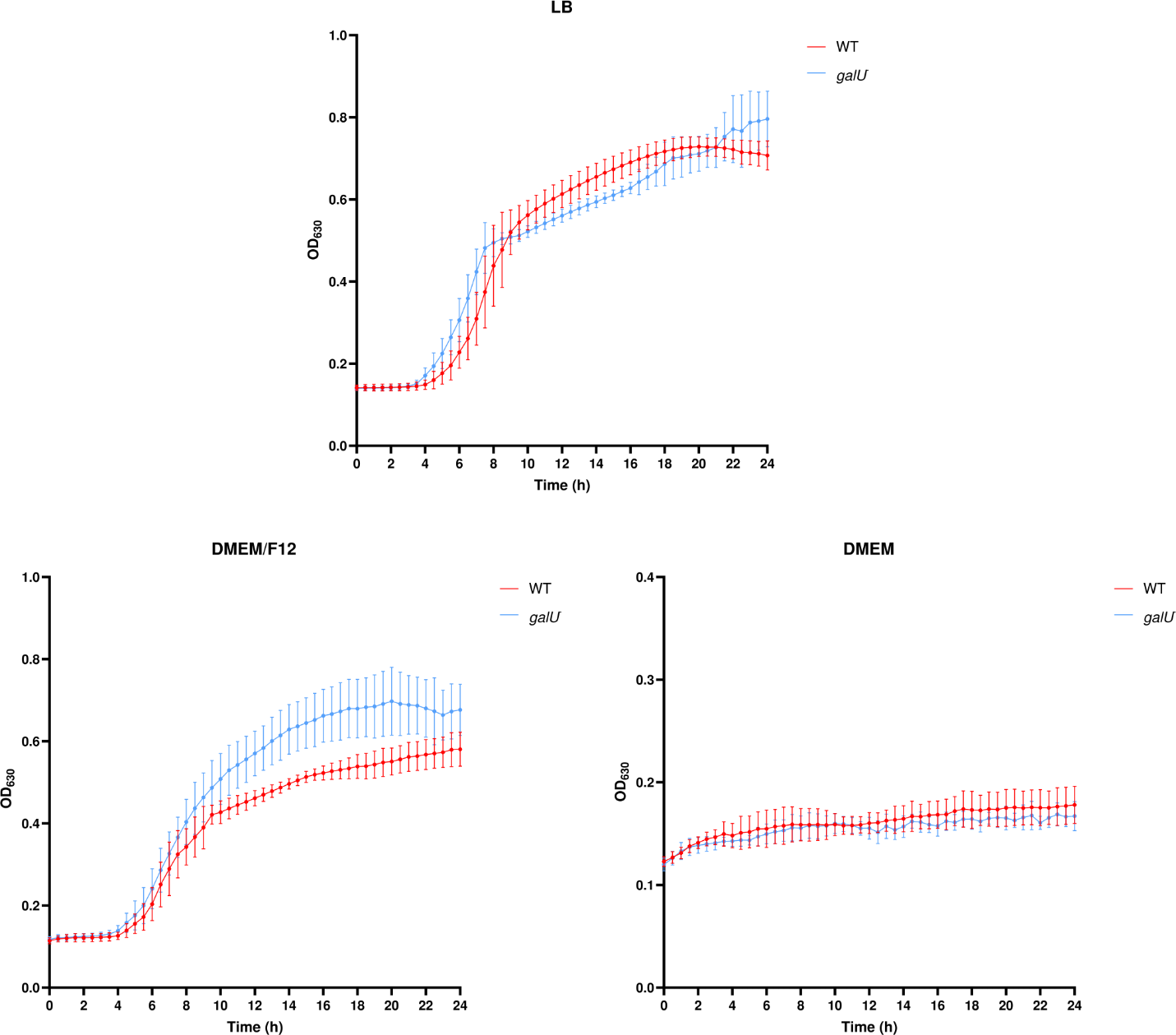


Fig. S1 Growth kinetics of PAO1 WT and galU^-^ over 24 h in LB medium, DMEM/F12 and DMEM**.** PAO1 WT and galU^-^ cultures reached maximum densities of 0.7 and 0.8 in LB (overnight culture medium), 0.6 and 0.7 in DMEM/F12 (PCLS medium), and 0.2 in DMEM (Calu-3 culture medium), respectively. Three individual experiments (i.e. inoculation of individual liquid cultures with different aliquots of bacteria from cryo-stock) were performed and OD was measured in technical triplicate every 30 min. Datapoints represent means of the 3 experiments ± SD.


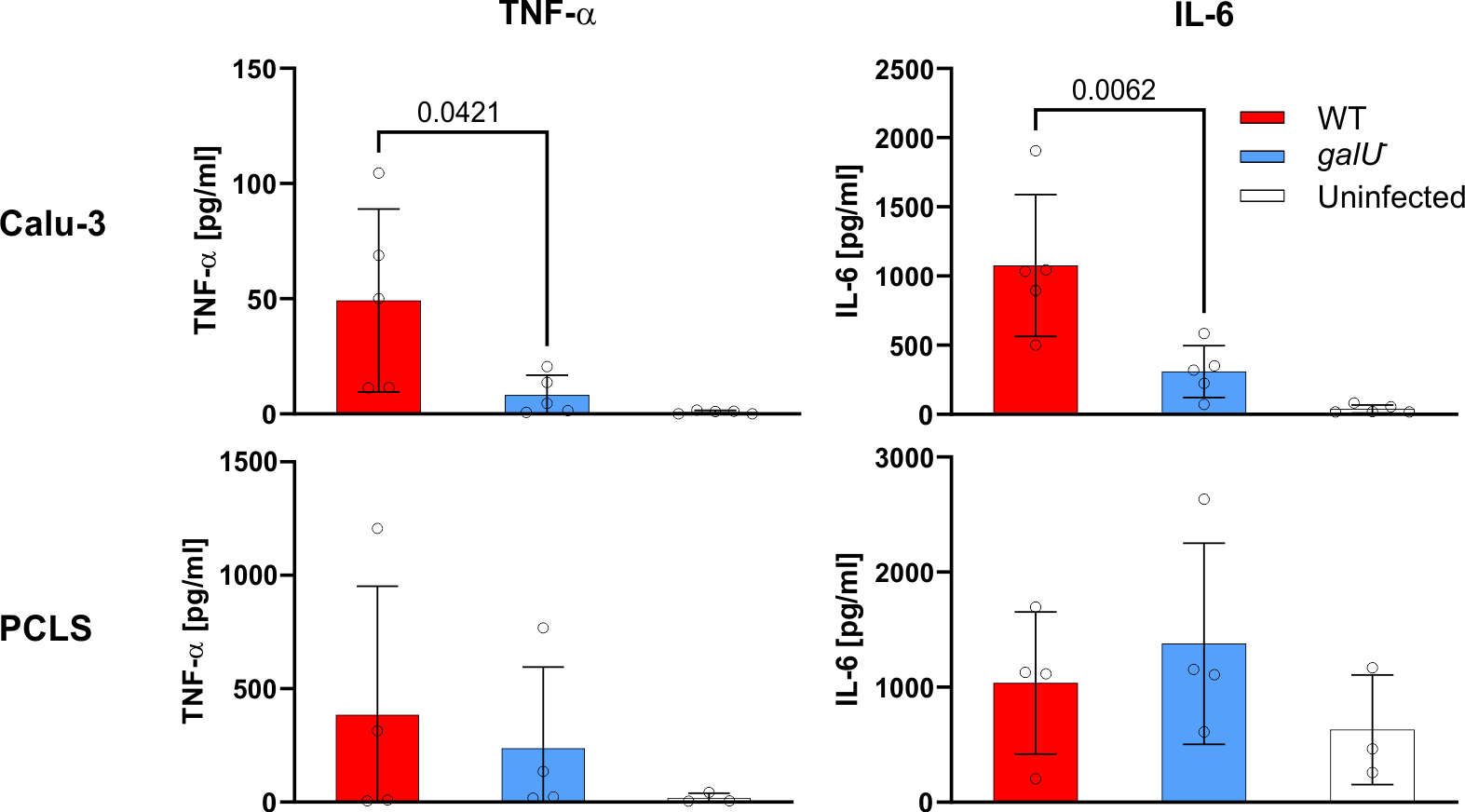


Fig. S2 Pro-inflammatory cytokines in culture supernatants of Calu-3 cells and PCLS. Secreted TNF-α and IL-6 were quantified by multiplex ELISA 6 h post-infection with PAO1 WT or galU^-^. Data from five (Calu-3) or four (PCLS) individual experiments. Error bars indicate mean ± SD. Adjusted p-values above bars indicate statistical significance determined by one-way ANOVA with Tukey’s multiple comparisons test.


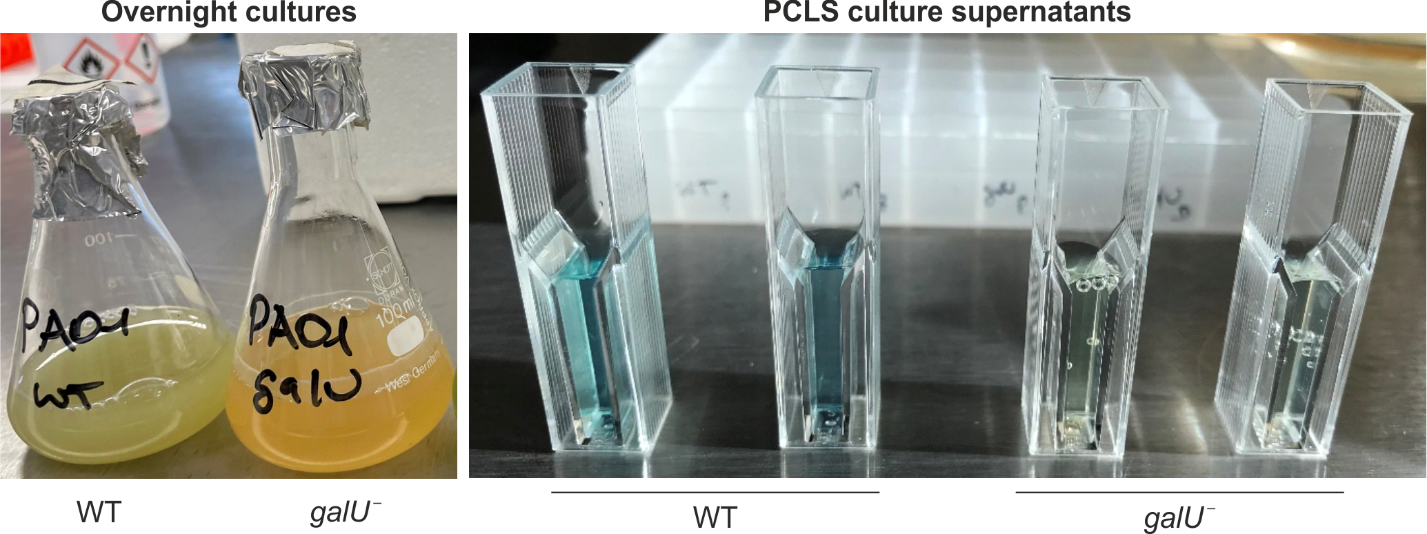


Fig. S3 Different pyocyanin levels in PAO1 WT and *galU^-^* culture supernatants. Left image: overnight cultures of PAO1 WT and *galU^-^*. Right image: cuvettes containing supernatants of PCLS infected with either WT or *galU^-^*.


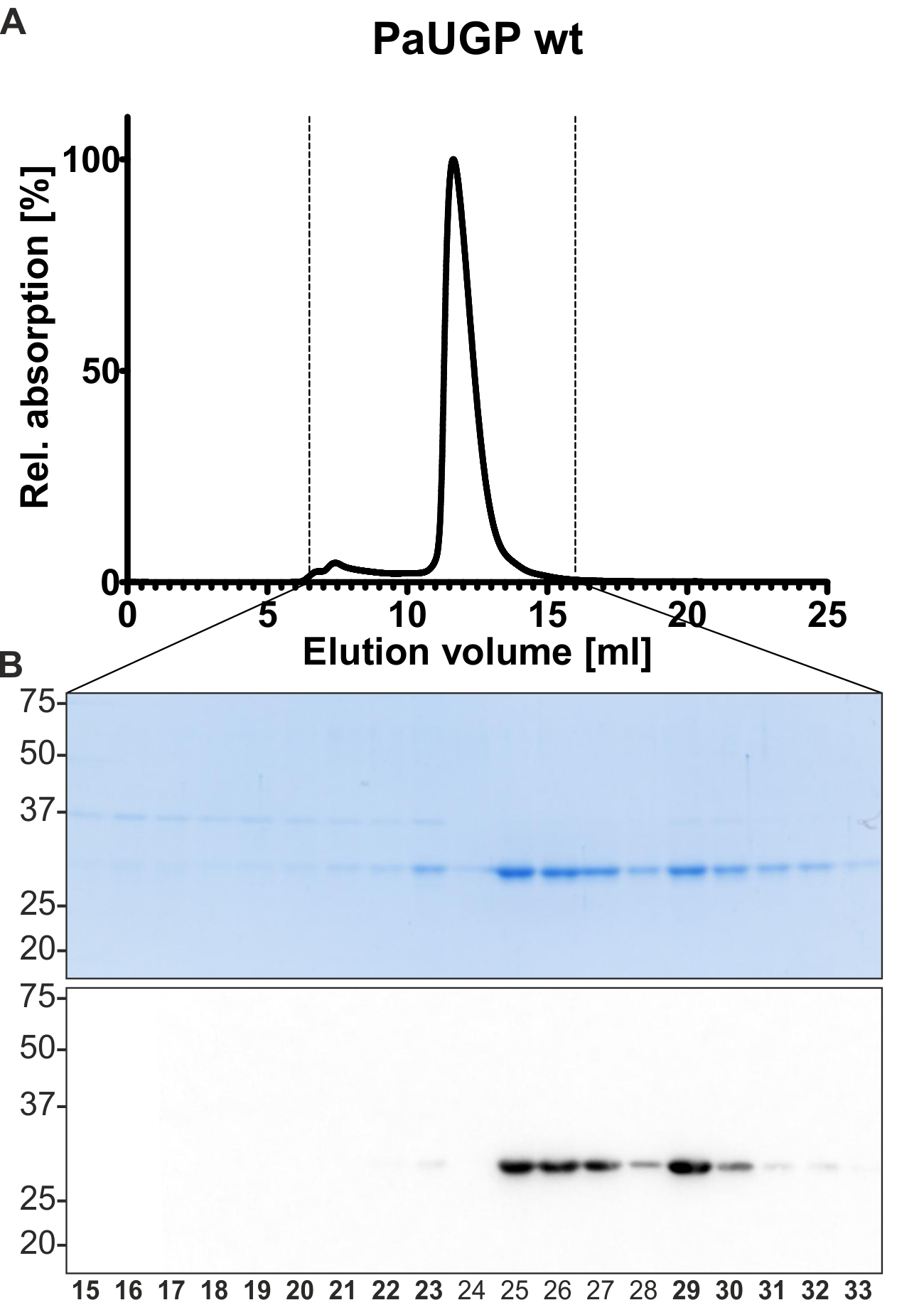


Fig. S4 Analysis of PaUGP oligomerization state and identification of the enzyme via SDS-PAGE and Western Blot. (A) Size exclusion chromatography profile of wild‑type PaUGP. (B) SDS-PAGE analysis of collected fractions. Top: Coomassie staining, bottom: Western Blot detecting the Strep-tag. Peak fractions 24-28 were diluted 1:10 (indicated by non-bold font).


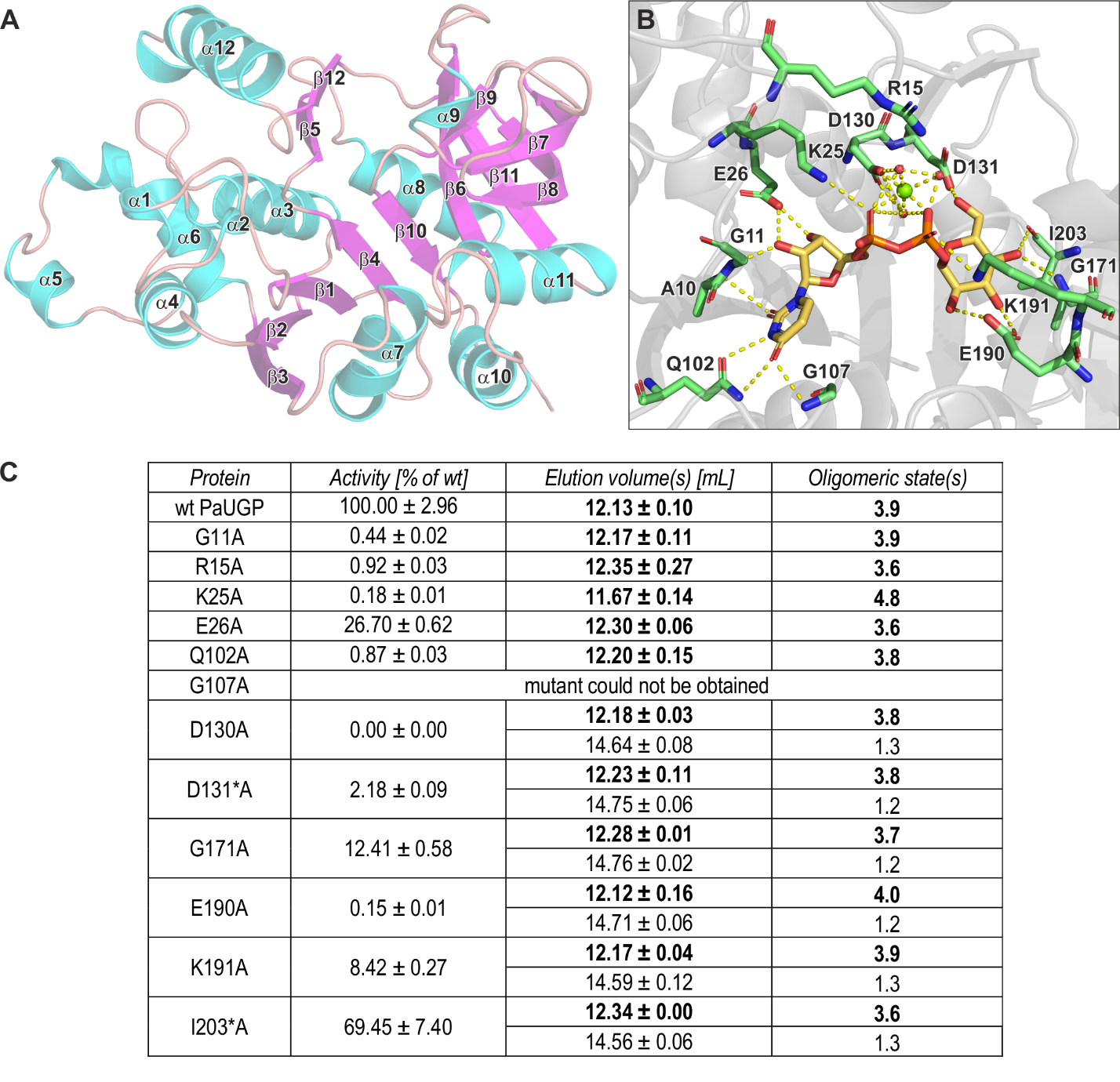


Fig. S5 PaUGP subunit structure and mutational analysis of active site. (A) Cartoon representation of the PaUGP secondary structure. α-helices, β-strands and loops are colored in cyan, magenta and light pink, respectively. (B) Close-up view of the active site with bound UDP-Glc and Mg^2+^. Key active site residues (lime), UDP-Glc (yellow), Mg^2+^ ion (green) and three water molecules (red) are shown in stick representation, with oxygen, nitrogen and phosphorus shown in red, blue and orange, respectively. Interactions are presented as yellow dotted lines*.* (C) Enzymatic activity and oligomeric state of PaUGP active site mutants. Activities were determined *in vitro* in the forward reaction and are given as means ± SEM of at least three individual experiments, each performed in technical triplicate, and expressed as % of wt activity which was defined as 100%. SEC elution volumes (compare Fig. S7) are given as means ± SD of at least two individual experiments. Oligomerization states were calculated based on elution volumes of proteins of known size. Where multiple protein peaks were observed, the main peak is given in bold print. *Amino acids not strictly conserved across bacterial UGPs, compare Fig. S8.


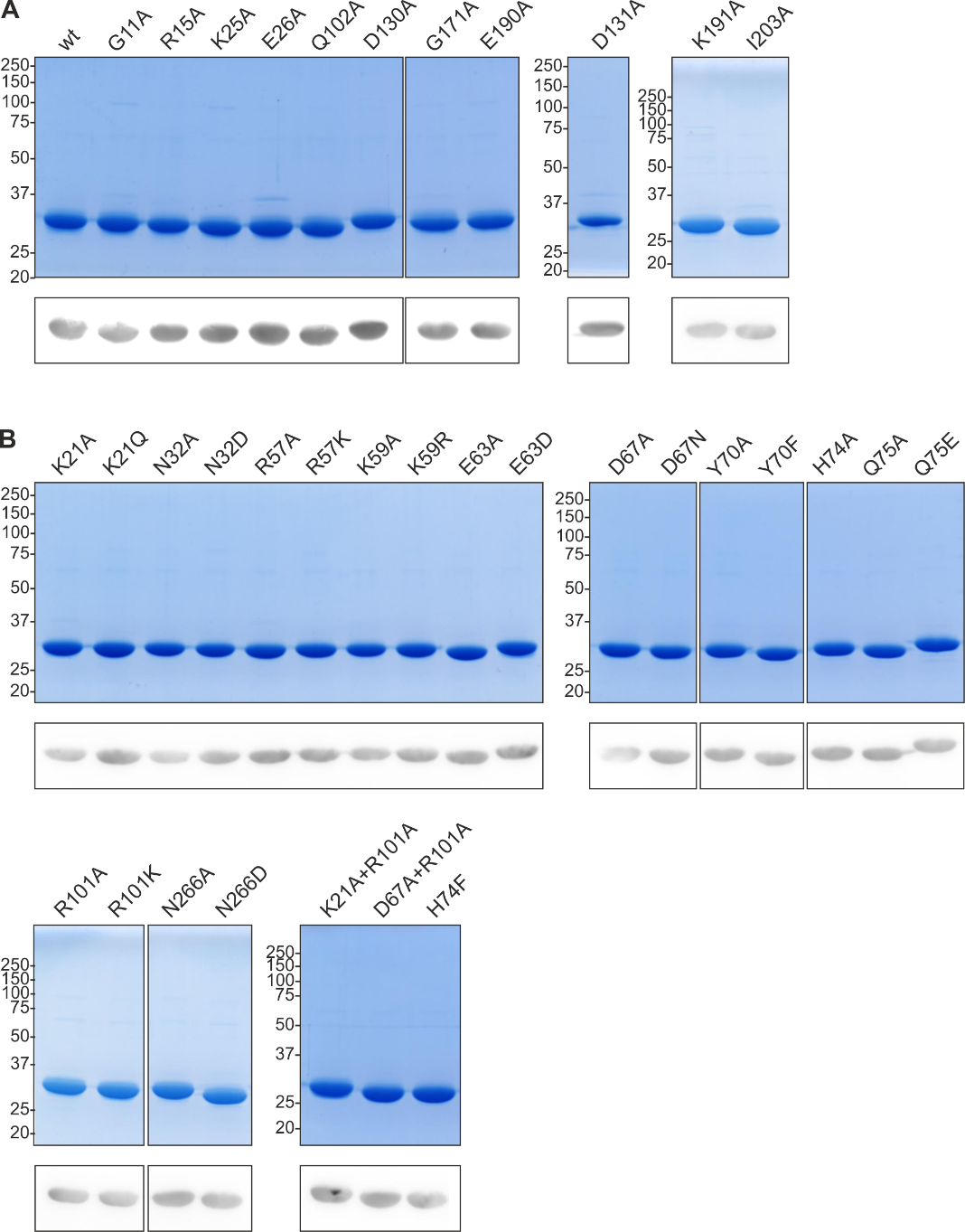


Fig. S6 SDS-PAGE analysis of purified recombinant Strep-tagged wild-type and mutant PaUGP. (A) PaUGP wt and active site mutants, (B) single and double mutants of proposed oligomerization residues. Top row, Coomassie staining; bottom row, Western Blot detecting the Strep-tag.


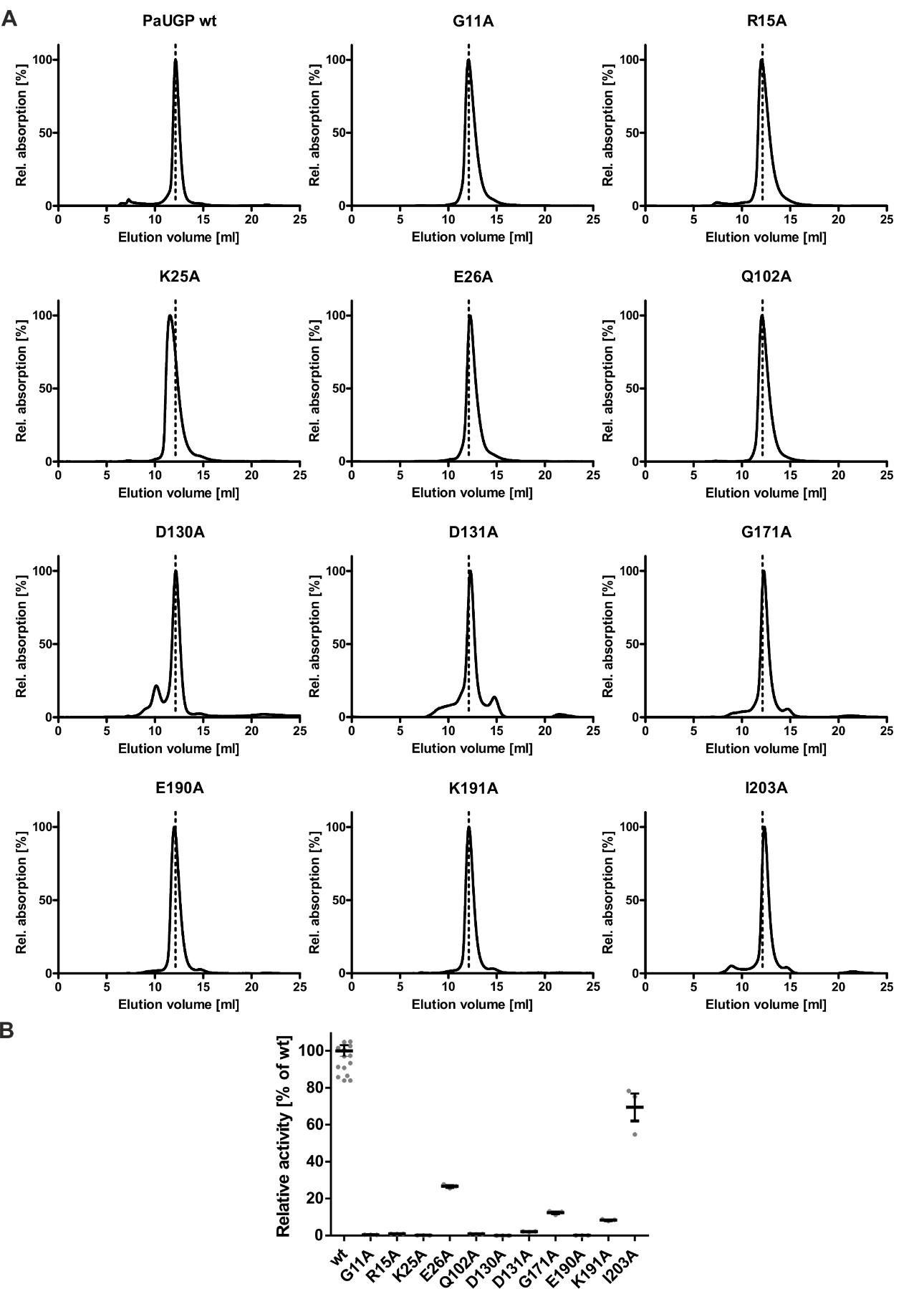


Fig. S7 Oligomeric states and activities of PaUGP wt and active site mutants in solution (compare Fig. S5C). (A) Size exclusion chromatography profiles of PaUGP active site mutants. For uniform presentation, relative absorption is depicted on the y-axis, with the main peak of each protein set to 100% of peak intensity. The dashed vertical line indicates the elution volume of tetrameric wt PaUGP for reference. (B) Relative activities of PaUGP active site mutants, depicted as means ± SEM of at least three individual experiments, each performed in technical triplicate.


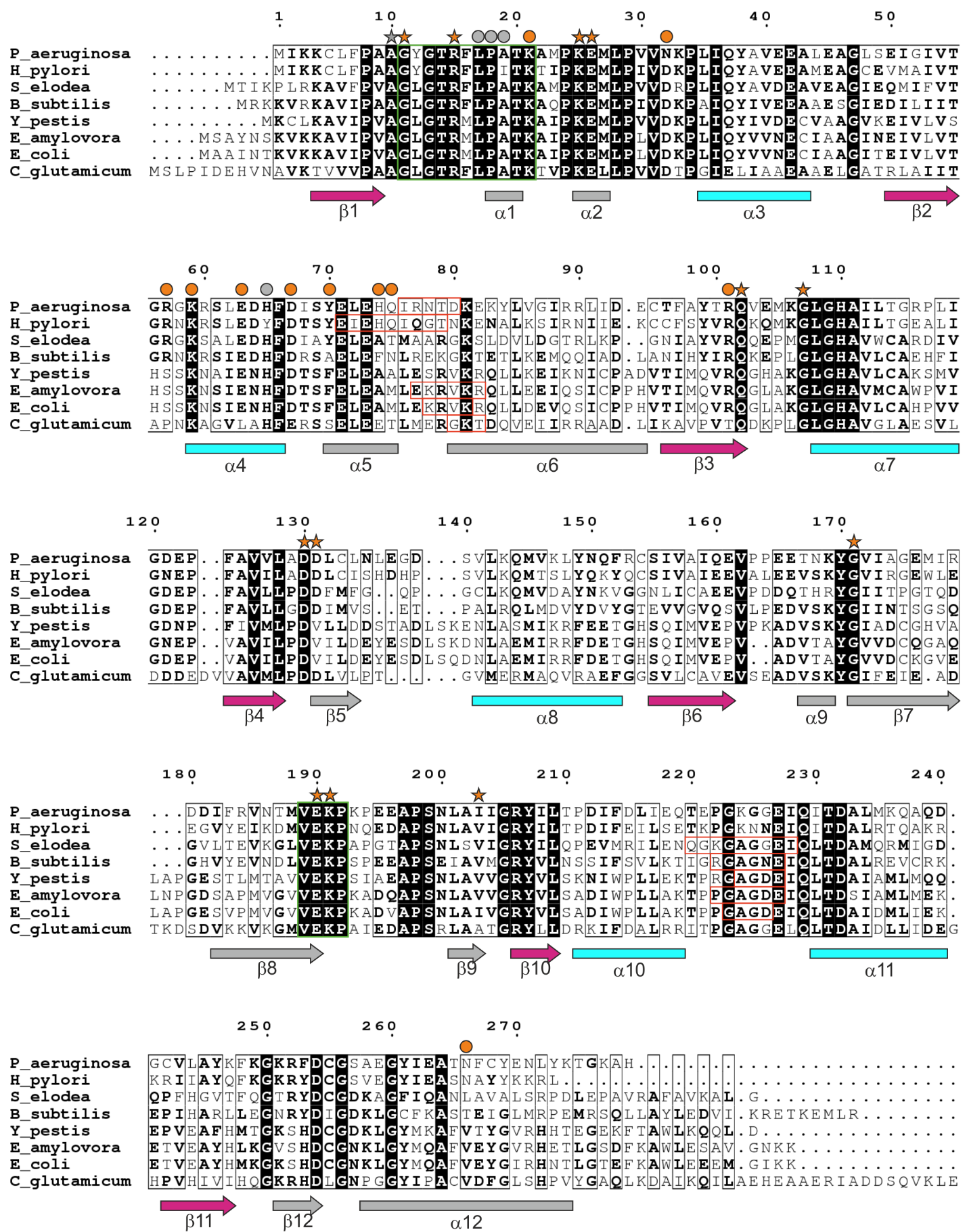


Fig. S8 Multiple sequence alignment of bacterial UGPs. PaUGP (this study) was aligned (using MultAlin (1)) with sequences of UGP from Helicobacter pylori, Sphingomonas elodea, Bacillus subtilis, Yersinia pestis, Erwinia amylovora, Escherichia coli and Corynebacterium glutamicum, whose structures have been solved and described in literature. Arrows and boxes denote β‑strands and α‑helices, respectively. α-helices and β-strands participating in the formation of the active site are highlighted in cyan and pink, respectively (compare Fig. S5A). Stars mark proposed active site residues and circles denote residues proposed to form intermolecular contacts, with positions mutated in this study highlighted in orange. Motifs conserved across bacterial UGPs are boxed in green. Red boxes indicate unresolved regions.


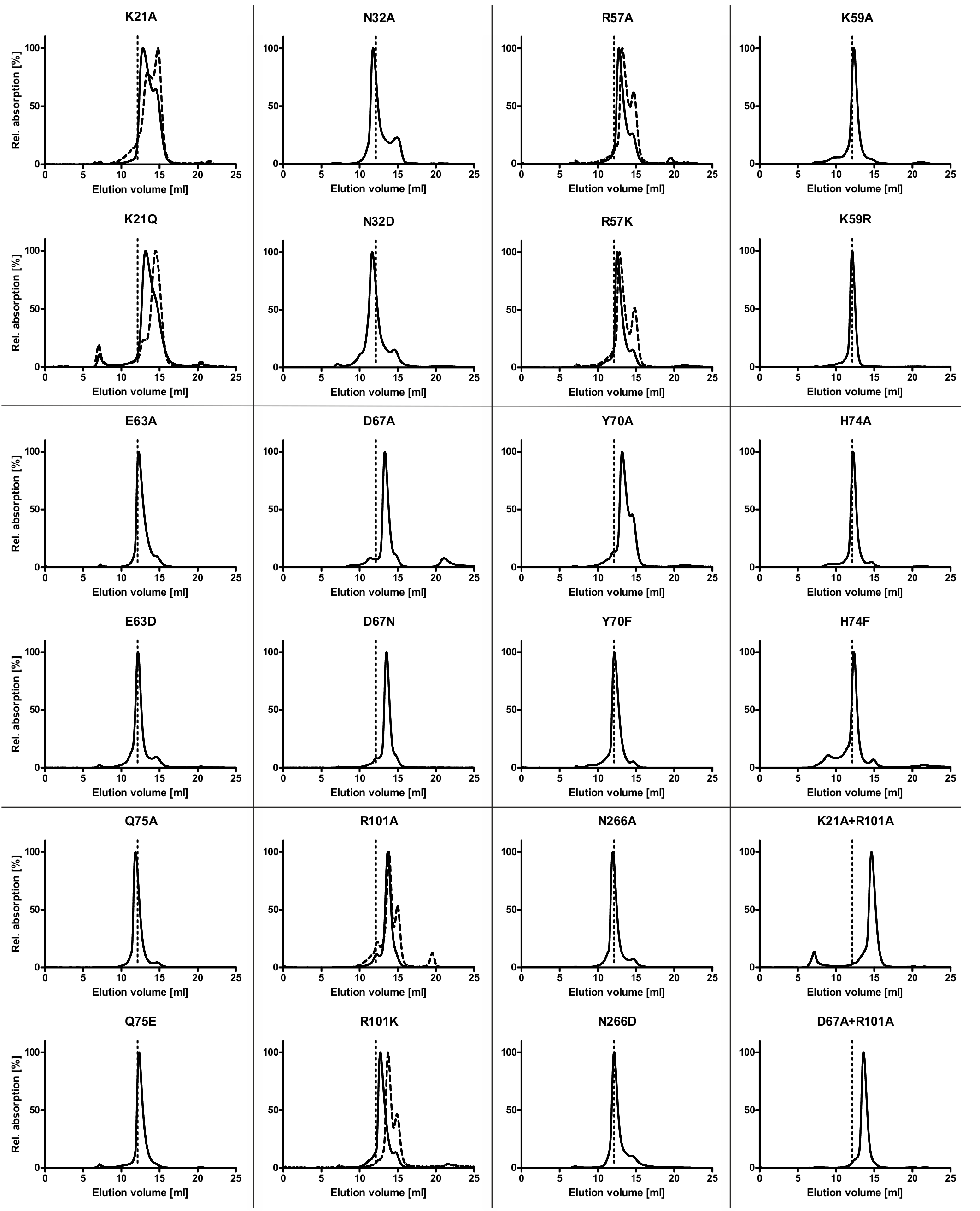


Fig. S9 Size exclusion chromatography profiles of PaUGP mutants of potential oligomerization-relevant residues (compare Table 1 of the main manuscript). For uniform presentation, relative absorption is depicted on the y-axis, with the main peak of each protein set to 100% of peak intensity. For mutants that show dilution-dependent dissociation, the elution profile of the more diluted sample is shown as a broken line. The dashed vertical line indicates the elution volume of tetrameric wt PaUGP for reference.


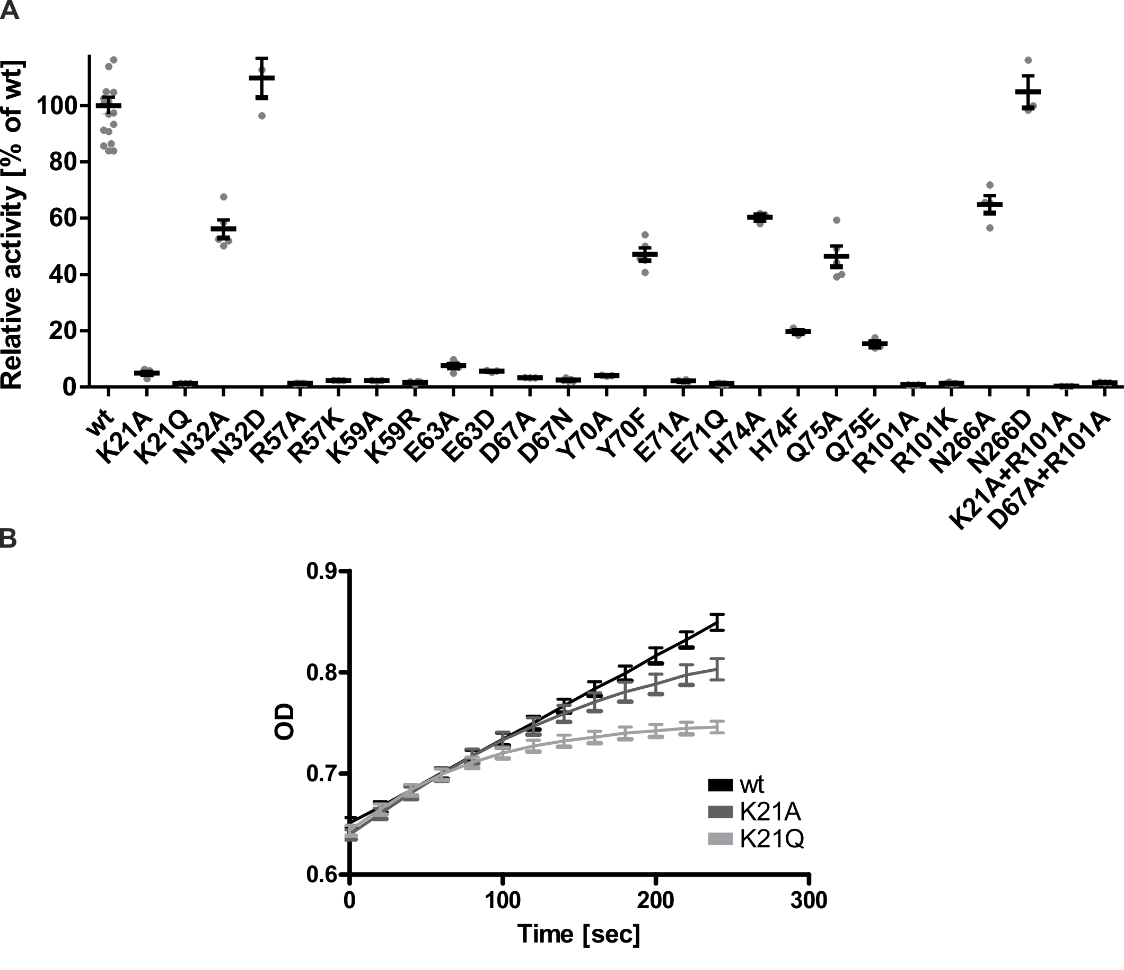


Fig. S10 Activities of PaUGP mutants of potential oligomerization-relevant residues. *In vitro* activity (forward reaction) was quantified using the EnzChek assay. (A) Relative activities of PaUGP mutants (compare Table 1 of the main manuscript), depicted as means ± SEM of at least three individual experiments, each performed in technical triplicate. (B) Progressive loss of *in vitro* activity of K21 mutants over time compared to wt PaUGP.


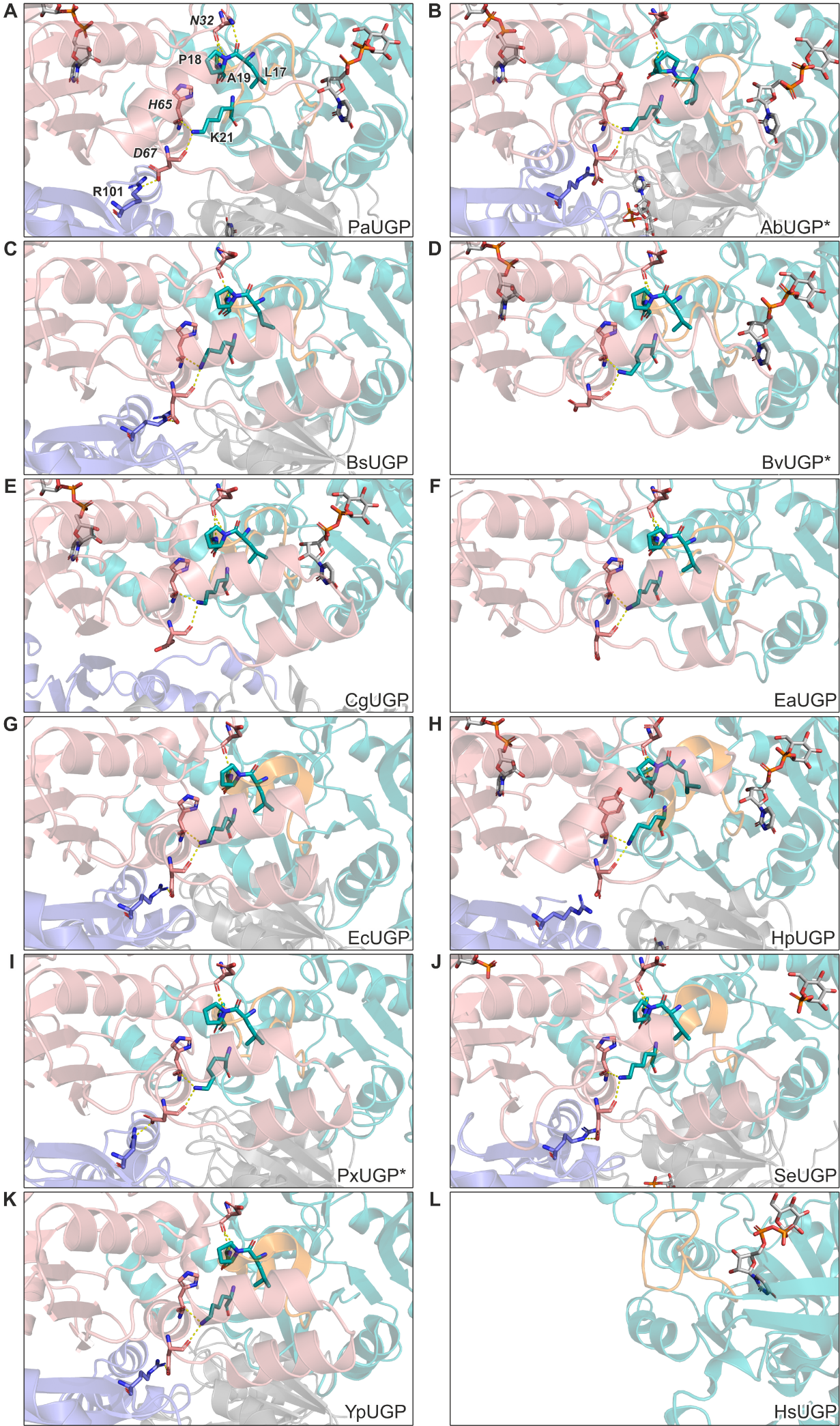


Fig. S11 Conservation of intermolecular interactions across the tight dimer interface (pink and cyan subunits) and (if applicable) the loose dimer interface (pink and purple subunit) in bacterial UGPs. (A) PaUGP in complex with UDP-Glc (this work), (B) *Acinetobacter baumannii* (Ab)UGP in complex with UDP-Glc (PDB 6K8D*), (C) *Bacillus subtilis* (Bs)UGP (PDB 7B1R / 7O2N), (D) *Burkholderia vietnamiensis* (Bv)UGP in complex with UDP-Glc (PDB 5I1F*), (E) *Corynebacterium glutamicum* (Cg)UGP in complex UDP-Glc (PDB 2PA4), (F) *Erwinia amylovora* (Ea)UGP (PDB 4D48), (G) *Escherichia coli* (Ec)UGP (PDB 2E3D), (H) *Helicobacter pylori* (Hp)UGP in complex with UDP-Glc (PDB 3JUK), (I) *Paraburkholderia xenovorans* (Px)UGP (PDB 5J49*), (J) *Sphingomonas elodea* (Se)UGP in complex with Glc-1-P (PDB 2UX8), (K) *Yersinia pestis* (Yp)UGP (PDB 6MNU), (L) *Homo sapiens* (Hs)UGP in complex with UDP‑Glc (PDB 4R7P). Asterisks indicate structures deposited in the PDB database but not yet described in publications. The loop corresponding to PaUGP A10-K21 is highlighted in orange. PaUGP L17, P18, A19, K21, N32, H65, D67 and R101 and their homologs in other bacterial UGPs, as well as bound substrates/products, are shown in stick representation with phosphorus, nitrogen and oxygen in orange, blue and red, respectively. Interactions are presented as yellow dotted lines.
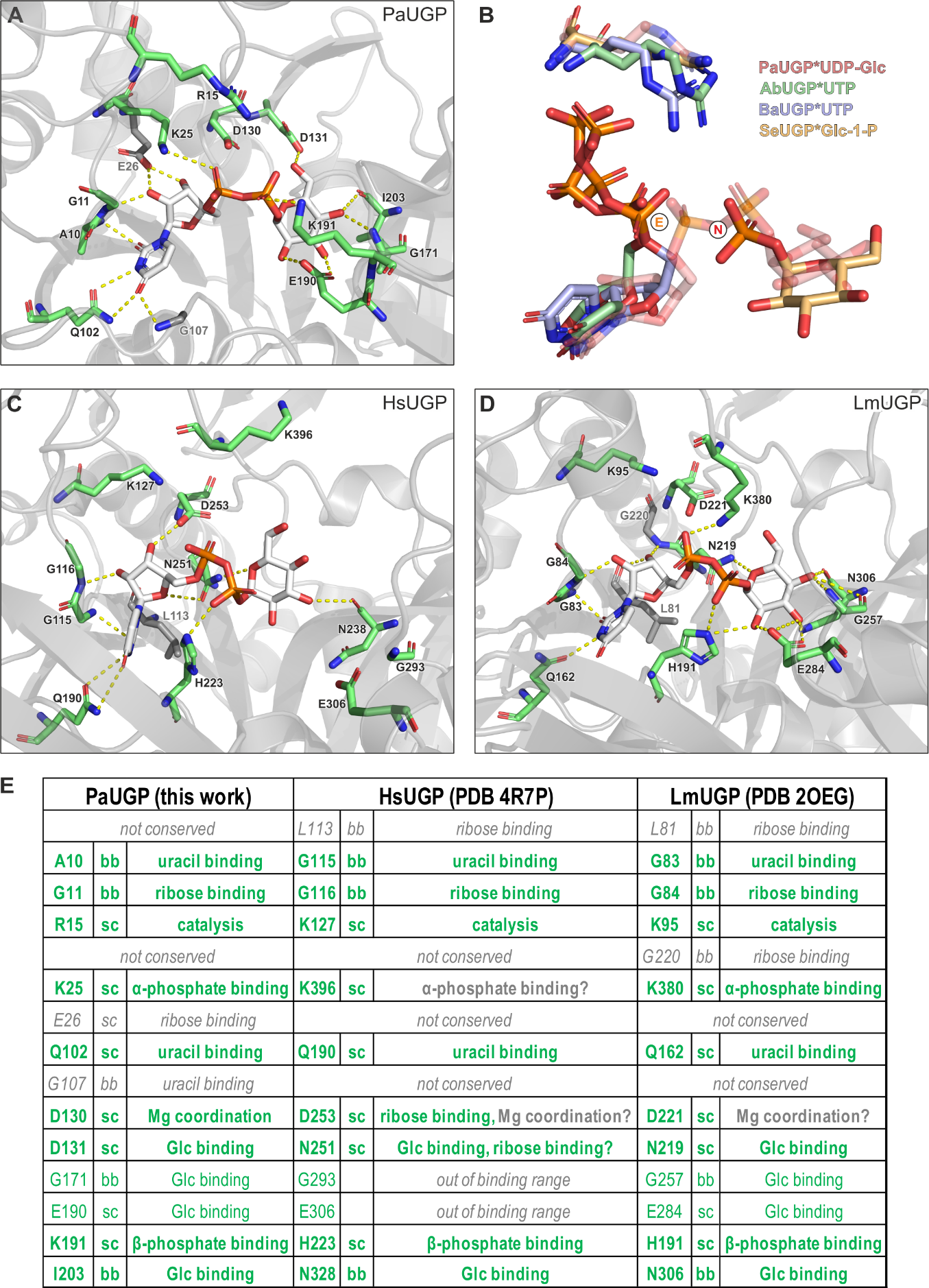


Fig. S12 Active sites of PaUGP, *Homo sapiens* UGP (HsUGP) and *Leishmania major* (LmUGP). (A) Active site of PaUGP (this work). (B) Proposed role of PaUGP R15 in substrate coordination. Pink, semi-transparent: R15 and UDP-Glc from PaUGP (this work); green: R14 and UTP from *Acinetobacter baumannii* (Ab)UGP (PDB 6KNJ); light blue: R17 and UTP from *Burkholderia ambifaria* (Ba)UGP (PDB 5VE7); yellow: R19 and Glc‑1‑P from *Sphingomonas elodea* (Se)UGP (PDB 2UX8). Phosphorus, oxygen and nitrogen shown in orange, red and blue, respectively. N and E denote the nucleophile and electrophile between which the UGP-catalyzed S_N_2 reaction occurs. (C) Active site of HsUGP (PDB 4R7P). (D) Active site of LmUGP (PDB 2OEG). In panels (A), (C), (D), residues interacting with UDP-Glc and/or known to be crucial for activity are shown as sticks, with strictly or functionally conserved residues depicted in green and non-conserved residues in grey. Nitrogen, oxygen and phosphorus are shown in blue, red and orange, respectively. Interactions are presented as yellow dotted lines. (E) Overview table of PaUGP, HsUGP and LmUGP active site residues and their roles in UGP function. Conserved interactions are printed in green; bold font indicates conserved function in all three enzymes. Sc, sidechain; bb, backbone.

Supplemental tables

Table S1 Bacterial loads in cell/tissue lysates and culture supernatants. Values are means of technical replicates from five individual experiments.

|  |  | **WT** | ***galU****^-^* |
| --- | --- | --- | --- |
| **Calu-3** | **Lysate** | 3.91E+07 | 1.13E+06 |
|  |  | 4.87E+07 | 5.80E+06 |
|  |  | 2.47E+07 | 6.50E+06 |
|  |  | 3.00E+07 | 1.37E+06 |
|  |  | 1.23E+08 | 8.53E+06 |
|  | **Supernatant** | 3.75E+08 | 1.13E+07 |
|  |  | 3.05E+08 | 8.80E+08 |
|  |  | 2.98E+08 | 5.40E+08 |
|  |  | 3.00E+08 | 3.00E+08 |
|  |  | 2.16E+09 | 1.10E+09 |
| **PCLS** | **Lysate** | 7.21E+07 | 1.27E+07 |
|  |  | 7.32E+07 | 1.73E+06 |
|  |  | 1.83E+07 | 5.28E+06 |
|  |  | 2.48E+07 | 1.10E+07 |
|  |  | 4.50E+05 | 7.67E+05 |
|  | **Supernatant** | 4.80E+09 | 1.04E+10 |
|  |  | 4.38E+09 | 2.30E+09 |
|  |  | 3.57E+09 | 2.83E+09 |
|  |  | 4.12E+09 | 3.85E+09 |
|  |  | 3.90E+09 | 3.40E+09 |

Table S2 Crystallographic statistics of PaUGP in complex with UDP-Glc**.** The statistics were calculated using AIMLESS (2) and MolProbity (3). Statistics for the highest-resolution shell are shown in parentheses. R_merge_ = ∑hkl∑j|Ihkl,j−⟨Ihkl⟩|/(∑hkl∑jIhkl,j). R_work_ / R_free_ = ∑hkl|Fhklobs−Fhklcalc|/(∑hklFhklobs); R_free_ was calculated using randomly chosen 5% fraction of data that was excluded from refinement.

| **PaUGP in complex with UDP-Glc** | |
| --- | --- |
| **Data Collection** | |
| Beamline | MX2, Australian Synchrotron |
| Number of images | 3600 |
| Detector | Dectris Eiger 16M |
| Detector distance | 269.39 |
| Wavelength (Å) | 0.9537 |
| Start angle (º) | 180 |
| Space group | P12_1_1 |
| Cell parameters  a, b, c (Å)  α, β, γ (°) | 84.71, 114.85, 155.76  90, 97.39, 90 |
| Resolution range (Å) | 46.98 – 2.9 (2.97 – 2.9) |
| No. of observations | 256,604 (17,662) |
| No. of unique observations | 65,549 (4608) |
| Completeness (%) | 99.7 (97.8) |
| Multiplicity | 3.9 (3.8) |
| Mean Ι/σ(I) | 9.3 (1.8) |
| R_merge_ | 0.109 (0.860) |
| R_meas_ | 0.148 (1.169) |
| R_pim_ | 0.099 (0.785) |
| CC1/2 | 0.995 (0.712) |
| **Refinement** | |
| Resolution (Å) | 2.9 |
| Number of reflections used | 65,443 |
| R-work | 0.195 |
| R-free | 0.236 |
| Clashscore | 6.11 |
| **Final Model** | |
| Number of protein residues | 1936 |
| Number of water molecules | 102 |
| Number of ligand atoms | 480 |
| Average B-factor (Å2) | 55.16 |
| Solvent content (%) | 58.33 |
| **R.M.S Deviations** | |
| Bond lengths (Å) | 0.0073 |
| Bond angles (º) | 0.997 |
| **Ramachandran plot (%)** | |
| Outliers (%) | 0.69 |
| Allowed (%) | 4.64 |
| Favoured (%) | 94.67 |

Table S3 Mutagenesis primers and their sequences**.** Altered bases are printed in lowercase; fw, forward; rev, reverse.

| **Description** | **Primer Sequence (5‘-3‘)** |
| --- | --- |
| PaUGP G11A fw | CCGCCGcTTACGGCACCCGTTTCCTCCCCG |
| PaUGP G11A rev | CCGTAAgCGGCGGCCGGGAAAAGACATTTCTTGATGGATC |
| PaUGP R15A fw | TACGGCACCgcTTTCCTCCCCGCCACCAAGGCCATG |
| PaUGP R15A rev | GGGGAGGAAAgcGGTGCCGTAACCGGCGGCC |
| PaUGP K21A fw | GCCACCgcgGCCATGCCCAAGGAAATGCTGCCGGTGG |
| PaUGP K21A rev | CATGGCcgcGGTGGCGGGGAGGAAACGGGTGCC |
| PaUGP K21Q fw | CCCCGCCACCcagGCCATGCCCAAGGAAATGCTGCCG |
| PaUGP K21Q rev | GGGCATGGCctgGGTGGCGGGGAGGAAACGGGTGC |
| PaUGP K25A fw | GCCATGCCCgcGGAAATGCTGCCGGTGGTGAACAAG |
| PaUGP K25A rev | CAGCATTTCCgcGGGCATGGCCTTGGTGGCGGGG |
| PaUGP E26A fw | ATGCCCAAGGcAATGCTGCCGGTGGTGAACAAGCCG |
| PaUGP E26A rev | CGGCAGCATTgCCTTGGGCATGGCCTTGGTGGC |
| PaUGP N32A fw | GGTGgccAAGCCGCTGATCCAGTACGCGGTGGAGG |
| PaUGP N32A rev | CAGCGGCTTggcCACCACCGGCAGCATTTCCTTGGG |
| PaUGP N32D fw | CCGGTGGTGgacAAGCCGCTGATCCAGTACGCGGTG |
| PaUGP N32D rev | CAGCGGCTTgtcCACCACCGGCAGCATTTCCTTGGGC |
| PaUGP R57A fw | GTCACCGGCgccGGCAAGCGTTCGCTGGAAGACCAC |
| PaUGP R57A rev | CGCTTGCCggcGCCGGTGACGATGCCGATCTCGGAA |
| PaUGP R57K fw | GTCACCGGCaagGGCAAGCGTTCGCTGGAAGACCAC |
| PaUGP R57K rev | CGCTTGCCcttGCCGGTGACGATGCCGATCTCGGAA |
| PaUGP K59A fw | GCgcGCGTTCGCTGGAAGACCACTTCGACA |
| PaUGP K59A rev | AGCGAACGCgcGCCGCGGCCGGT |
| PaUGP K59R fw | GCAgGCGTTCGCTGGAAGACCACTTCGACA |
| PaUGP K59R rev | AGCGAACGCctGCCGCGGCCGGT |
| PaUGP E63A fw | GTTCGCTGgcaGACCACTTCGACATCAGCTACGAGCTG |
| PaUGP E63A rev | GGTCtgcCAGCGAACGCTTGCCGCGGCC |
| PaUGP E63D fw | CGTTCGCTGgacGACCACTTCGACATCAGCTACGAGC |
| PaUGP E63D rev | GAAGTGGTCgtcCAGCGAACGCTTGCCGCGGC |
| PaUGP D67A fw | GACCACTTCgccATCAGCTACGAGCTGGAACACCAG |
| PaUGP D67A rev | GTAGCTGATggcGAAGTGGTCTTCCAGCGAACGC |
| PaUGP D67N fw | CACTTCaacATCAGCTACGAGCTGGAACACCAGATC |
| PaUGP D67N rev | GCTGATgttGAAGTGGTCTTCCAGCGAACGCTTG |
| PaUGP Y70A fw | CATCAGCgcCGAGCTGGAACACCAGATCCGCAACAC |
| PaUGP Y70A rev | CAGCTCGgcGCTGATGTCGAAGTGGTCTTCCAGCGAAC |
| PaUGP Y70F fw | CATCAGCTtCGAGCTGGAACACCAGATCCGCAACAC |
| PaUGP Y70F rev | CAGCTCGaaGCTGATGTCGAAGTGGTCTTCCAGCGAAC |
| PaUGP H74A fw | CTGGAAgcCCAGATCCGCAACACCGACAAGG |
| PaUGP H74A rev | GATCTGGgcTTCCAGCTCGTAGCTGATGTCGAAGTG |
| PaUGP H74F fw | CTGGAAttCCAGATCCGCAACACCGACAAG |
| PaUGP H74F rev | GATCTGGaaTTCCAGCTCGTAGCTGATGTCG |
| PaUGP Q75A fw | GGAACACgcgATCCGCAACACCGACAAGGAAAAGTACCTGG |
| PaUGP Q75A rev | CGGATcgcGTGTTCCAGCTCGTAGCTGATGTCGAAGTGG |
| PaUGP Q75E fw | CTGGAACACgagATCCGCAACACCGACAAGGAAAAGTAC |
| PaUGP Q75E rev | GTTGCGGATctcGTGTTCCAGCTCGTAGCTGATGTCGA |
| PaUGP R101A fw | GCCTACACCgccCAGGTGGAGATGAAGGGCCTCGG |
| PaUGP R101A rev | CTCCACCTGcgcGGTGTAGGCGAAGGTGCACTCGTC |
| PaUGP R101K fw | CTACACCaagCAGGTGGAGATGAAGGGCCTCG |
| PaUGP R101K rev | CCTGcttGGTGTAGGCGAAGGTGCACTCGTC |
| PaUGP Q102A fw | TACACCCGCgcGGTGGAGATGAAGGGCCTCGGC |
| PaUGP Q102A rev | CATCTCCACCgcGCGGGTGTAGGCGAAGGTGCAC |
| PaUGP D130A fw | GTCCTGGCCGcCGACCTGTGCCTGAACCTCGAAGGCG |
| PaUGP D130A rev | GCACAGGTCGgCGGCCAGGACCACGGCGAACGG |
| PaUGP D131A fw | CCGACGcCCTGTGCCTGAACCTCGAAGGCG |
| PaUGP D131A rev | CACAGGgCGTCGGCCAGGACCACGGCG |
| PaUGP G171A fw | CAAGTACGcCGTGATCGCCGGCGAGATGATCCG |
| PaUGP G171A rev | GATCACGgCGTACTTGTTGGTCTCTTCCGGCGG |
| PaUGP E190A fw | ACCATGGTCGcGAAGCCGAAGCCGGAAGAGGCGC |
| PaUGP E190A rev | CTTCGGCTTCgCGACCATGGTGTTCACCCGGAAGATGTC |
| PaUGP K191A fw | ATGGTCGAGgcGCCGAAGCCGGAAGAGGCGCCGT |
| PaUGP K191A rev | CGGCTTCGGCgcCTCGACCATGGTGTTCACCCGGAAG |
| PaUGP I203A fw | CTGGCGgcCATCGGCCGCTACATCCTGACCCC |
| PaUGP I203A rev | CCGATGgcCGCCAGGTTCGACGGCGCCTCTTC |
| PaUGP N266A fw | GCGACCgccTTCTGCTACGAAAACCTCTACAAGACCGGC |
| PaUGP N266A rev | GCAGAAggcGGTCGCCTCGATGTAACCCTCGGCG |
| PaUGP N266D fw | GAGGCGACCgacTTCTGCTACGAAAACCTCTACAAGACCG |
| PaUGP N266D rev | GTAGCAGAAgtcGGTCGCCTCGATGTAACCCTCGGC |

Supplemental discussion

### Changes in pigment production

*P. aeruginosa* produces several pigments including the phenazine pyocyanin which has been implicated in pathogenesis and is considered a major virulence factor as it can cross membranes and cause oxidative stress in host cells (4, 5). Pyocyanin produced by PAO1 *galU^-^* was significantly reduced compared to WT cultures (Fig. 2A-C, hatched bars). Interestingly, contact with Calu-3 cells increased pyocyanin production significantly in PAO1 WT, but not in PAO1 *galU^-^*, although a similar trend was seen there (Fig. 2C). The decrease in secreted pyocyanin observed in the *galU*-deficient mutant could be explained through regulatory effects of UDP-Glc on expression of the alternative sigma factor σ^s^ (also called RpoS), a transcriptional regulator activated at the onset of stationary phase growth. Overexpression of *rpoS* resulted in a decrease of pyocyanin in culture supernatants whereas an *rpoS*-deficient mutant produced twice as much pyocyanin as the parent strain PAO1 (6). UDP-Glc is a negative regulator of *rpoS* expression in Gram-negative *E. coli* and mutation of *galU* leads to heightened RpoS levels in these bacteria (7). The absence of UDP-Glc in the PAO1 *galU^-^* mutant might similarly result in increased *rpoS* expression and thus repression of pyocyanin production.

Since incubation of Calu-3 cells with up to 128 µM pyocyanin (≈ 30 times higher than the pyocyanin levels measured in WT supernatants) did not induce any detectable cytotoxicity (Fig. 2D), the reduced virulence of the *galU^-^* mutant cannot be attributed to decreases in pyocyanin alone. Rather, we propose that in the native biological context, pyocyanin acts in concert with other virulence factors to exert its cytotoxic effects.

### Motility phenotype

*The* *galU^-^* mutant exhibited significantly reduced swimming motility and almost complete absence of swarming behavior compared to PAO1 WT (Fig. 3). This phenotype is in line with previous reports describing LPS-mediated changes in motility in different bacteria. Specifically, PAO1 mutants producing truncated LPS core oligosaccharide displayed defects in both swimming and swarming motility as well as stronger attachment to abiotic surfaces; likely because changes in cell-surface physicochemistry affected bacterial attachment to the substratum, and/or cell-cell interactions (8). Azimi and coworkers reported that loss of O-antigen and changes in the LPS core increased cell surface hydrophobicity and promoted bacterial clumping (9). Both these factors could similarly hinder effective outward migration of the PAO1 *galU^-^* mutant through motility assay media. O-antigen was also implied to facilitate swarming motility of *Salmonella enterica* by increasing hydration of the cell surface and the substratum due to its hydrophilicity (10) .

### PaUGP active site geometry and species comparisons

Comparison of our PaUGP*UDP-Glc complex crystal structure with published bacterial UGP structures reveals the secondary and tertiary structure as well as most active site residues to be highly conserved among bacterial UGPs. Major differences are found at the C-terminus, where UGPs from *Sphingomonas elodea* (Se) (11), *Bacillus subtilis* (Bs) (12), *Yersinia pestis* (Yp) (13), *Erwinia amylovora* (Ea) (14)*, Escherichia coli* (Ec) (15) and *Corynebacterium glutamicum* (Cg) (16) possess an additional α‑helix of 12 to 22 residues length that is absent from PaUGP and *Helicobacter pylori* (Hp) UGP (17), used here as model for molecular replacement (Fig. S8). Two sections of the protein backbone could not be fully traced in several published bacterial UGP structures (Fig. S8), the first corresponding to PaUGP I76-T79, which likewise could not be fully modelled in all PaUGP subunits. This region corresponds to a loop between PaUGP α5 and α6 located at the opening of the neighboring subunit’s catalytic center and has been previously described to act as a “back side lid” to the active site (14). Another region that was not fully resolved in Se, Bs, Yp, Ea and EcUGP corresponds to PaUGP loop T220-Q229 and contributes to the active site architecture, specifically the sugar binding pocket. This region could be traced exclusively in bacterial UGPs crystallized in the presence of UDP-Glc.

The uridine moiety of UDP-Glc engages in H-bonds with PaUGP A10, Q102 and G107 (uracil ring) as well as G11 and E26 (ribose ring) (Fig. S5B). Mutants Q102A and G11A were nearly inactive, whereas E26A exhibited around 27% of residual activity (Fig. S5C). Coordination of the glucose moiety is facilitated by G171 and I203, whose mutants exhibited ca. 12% and 70% of wt activity, respectively (Fig. S5C). I203 is not strictly conserved (compare Fig. S8) and interacts with the Glc 4-OH group via its backbone, which is likely the reason that its mutant exhibits comparatively high residual activity. The same backbone interaction, established by either an isoleucine or valine, can be seen in all other UGPs crystallized in presence of substrate/product. The glucose moiety is further coordinated by acidic residues D131 and E190, and mutation of either residue to alanine abolished activity almost entirely (Fig. S5C). E190 is part of the VEKP signature motif of bacterial UGPs (18) and establishes H-bonds to the hydroxyl groups in positions 2 and 3 of the glucose ring. D131, whose sidechain in our structure forms a H-bond with the Glc 6‑OH group, is replaced by valine in (among others) UGPs from *Erwinia amylovora* (Ea) and *Escherichia coli* (Ec) (Fig. S8). While the aforementioned H‑bond with Glc cannot be established by valine in the respective position, Benini and colleagues suggested that the shorter valine sidechain in this position could facilitate substrate promiscuity, as both the Ea and Ec UGP were able to utilize various sugar‑1‑phosphate as substrates (14).

The α- and β-phosphoryl groups of UDP-Glc engage in electrostatic interactions with K25 and K191, respectively, as well as the Mg^2+^ ion (Fig. S5B). While K191A displayed ca. 8% of wt activity, K25A was almost entirely inactive (Fig. S5C). Mg^2+^ is additionally coordinated by D130 and three water molecules and is thought to compensate the negative charges of the phosphoryl groups and to facilitate catalysis by correctly orienting the sugar-1-phosphate for its nucleophilic attack on the nucleoside triphosphate’s α-phosphate in bacterial UGPs (16) and related nucleotidyltransferases (19). In line with this crucial role of Mg^2+^ in catalysis, D130A had the most severe impact of all introduced mutations and led to a complete loss of activity (Fig. S5C). A putative catalytic residue, R15, was also mutated since it is part of the conserved GXGTRXLPXTK motif (20) (PaUGP G11 – K21) bordering the uridyl binding pocket of the active site, and is highly conserved across bacterial UGPs (20) and related nucleotidyltransferases (18, 21). In our structure, R15 was located too distant from UDP-Glc to be involved in product binding, but was essential for activity (Fig. S5C). In the related Glc-1-P thymidylyltransferase of *P. aeruginosa*, the homologue of R15 was proposed to play a key role in catalysis by orienting the pyrophosphate group and/or compensating its charge and by binding the substrate desoxythymidintriphosphate in a strained conformation ideal for nucleophilic attack by Glc‑1‑P (22). Indeed, also in the crystal structures of *Acinetobacter baumannii* and *Burkholderia ambifaria* UGP (PDB 6KNJ and 5VE7; both to be published), which were solved in presence of UTP, the corresponding arginines clearly coordinate the UTP phosphate moiety. Superimposition of these structures with that of the SeUGP*Glc-1-P complex (PDB 2UX8) (11) reveals how the conserved arginine helps to bind UTP in a conformation that would enable the attack of Glc‑1-P (nucleophile) on the UTP α-phosphate (electrophile) (Fig. S12B).

References

1. Corpet F. 1988. Multiple sequence alignment with hierarchical clustering. Nucleic Acids Res 16:10881–10890.

2. Evans PR, Murshudov GN. 2013. How good are my data and what is the resolution? urn:issn:0907-4449 69:1204–1214.

3. Williams CJ, Headd JJ, Moriarty NW, Prisant MG, Videau LL, Deis LN, Verma V, Keedy DA, Hintze BJ, Chen VB, Jain S, Lewis SM, Arendall WB, Snoeyink J, Adams PD, Lovell SC, Richardson JS, Richardson DC. 2018. MolProbity: More and better reference data for improved all-atom structure validation. Protein Sci 27:293–315.

4. Gupte A, Jyot J, Ravi M, Ramphal R. 2021. High pyocyanin production and non-motility of Pseudomonas aeruginosa isolates are correlated with septic shock or death in bacteremic patients. PLoS One2021/06/12. 16:e0253259.

5. Hall S, McDermott C, Anoopkumar-Dukie S, McFarland AJ, Forbes A, Perkins A v., Davey AK, Chess-Williams R, Kiefel MJ, Arora D, Grant GD. 2016. Cellular effects of pyocyanin, a secreted virulence factor of Pseudomonas aeruginosa. Toxins (Basel) 8:1–14.

6. Suh SJ, Silo-Suh L, Woods DE, Hassett DJ, West SE, Ohman DE. 1999. Effect of rpoS mutation on the stress response and expression of virulence factors in Pseudomonas aeruginosa. J Bacteriol1999/06/29. 181:3890–3897.

7. Bohringer J, Fischer D, Mosler G, Hengge-Aronis R. 1995. UDP-glucose is a potential intracellular signal molecule in the control of expression of sigma S and sigma S-dependent genes in Escherichia coli. J Bacteriol1995/01/01. 177:413–422.

8. Lindhout T, Lau PCY, Brewer D, Lam JS. 2009. Truncation in the core oligosaccharide of lipopolysaccharide affects flagella-mediated motility in Pseudomonas aeruginosa PAO1 via modulation of cell surface attachment. Microbiology (N Y) 155:3449–3460.

9. Azimi S, Thomas J, Cleland SE, Curtis JE, Goldberg JB, Diggle SP. 2021. O-Specific Antigen-Dependent Surface Hydrophobicity Mediates Aggregate Assembly Type in Pseudomonas aeruginosa. mBio2021/08/11. 12:e0086021.

10. Toguchi A, Siano M, Burkart M, Harshey RM. 2000. Genetics of swarming motility in Salmonella enterica serovar typhimurium: critical role for lipopolysaccharide. J Bacteriol2000/10/29. 182:6308–6321.

11. Aragão D, Fialho AM, Marques AR, Mitchell EP, Sá-Correia I, Frazão C, Aragao D, Fialho AM, Marques AR, Mitchell EP, Sa-Correia I, Frazao C. 2007. The complex of Sphingomonas elodea ATCC 31461 glucose-1-phosphate uridylyltransferase with glucose-1-phosphate reveals a novel quaternary structure, unique among nucleoside diphosphate-sugar pyrophosphorylase members. J Bacteriol 189:4520–4528.

12. Wu CH, Rismondo J, Morgan RML, Shen Y, Loessner MJ, Larrouy-Maumus G, Freemont PS, Gründling A. 2021. Bacillus subtilis YngB contributes to wall teichoic acid glucosylation and glycolipid formation during anaerobic growth. Journal of Biological Chemistry 296:100384.

13. Gibbs ME, Lountos GT, Gumpena R, Waugh DS. 2019. Crystal structure of UDP-glucose pyrophosphorylase from Yersinia pestis, a potential therapeutic target against plague. Acta Crystallogr F Struct Biol Commun 75:608–615.

14. Benini S, Toccafondi M, Rejzek M, Musiani F, Wagstaff BA, Wuerges J, Cianci M, Field RA. 2017. Glucose-1-phosphate uridylyltransferase from Erwinia amylovora: Activity, structure and substrate specificity. Biochim Biophys Acta Proteins Proteom 1865:1348–1357.

15. Thoden JB, Holden HM. 2007. The molecular architecture of glucose-1-phosphate uridylyltransferase. Protein Science 16:432–440.

16. Thoden JB, Holden HM. 2007. Active site geometry of glucose-1-phosphate uridylyltransferase. Protein Sci 16:1379–1388.

17. Kim H, Choi J, Kim T, Lokanath NK, Ha SC, Suh SW, Hwang HY, Kim KK. 2010. Structural basis for the reaction mechanism of UDP-glucose pyrophosphorylase. Mol Cells 29:397–405.

18. Silva E, Marques AR, Fialho AM, Granja AT, Sá-Correia I. 2005. Proteins encoded by Sphingomonas elodea ATCC 31461 rmlA and ugpG genes, involved in gellan gum biosynthesis, exhibit both dTDP- and UDP-glucose pyrophosphorylase activities 71:4703–4712.

19. Sivaraman J, Sauve V, Matte A, Cygler M. 2002. Crystal structure of Escherichia coli glucose-1-phosphate thymidylyltransferase (RffH) complexed with dTTP and Mg2+. JBiolChem 277:44214–44219.

20. Marques AR, Ferreira PB, Sá-Correia I, Fialho AM. 2003. Characterization of the ugpG gene encoding a UDP-glucose pyrophosphorylase from the gellan gum producer Sphingomonas paucimobilis ATCC 31461. Molecular Genetics and Genomics 268:816–824.

21. Brown K, Pompeo F, Dixon S, Mengin-Lecreulx D, Cambillau C, Bourne Y. 1999. Crystal structure of the bifunctional N-acetylglucosamine 1-phosphate uridyltransferase from Escherichia coli: a paradigm for the related pyrophosphorylase superfamily. EMBO J 18:4096–4107.

22. Blankenfeldt W, Asuncion M, Lam JS, Naismith JH. 2000. The structural basis of the catalytic mechanism and regulation of glucose-1-phosphate thymidylyltransferase (RmlA). EMBO J 19:6652–63.
